# Co-targeting CDK4/6 and MEK reverses mesenchymal transition in therapy-refractory BRAF-altered pediatric high-grade glioma

**DOI:** 10.1186/s13046-026-03725-3

**Published:** 2026-05-16

**Authors:** Lisa Mayr, Leah Mager, Lisa Gabler-Pamer, Dominik Kirchhofer, Julia Freund, Kallen Schwark, Benison Lau, Sarah Machreich, Daniel Senfter, Mia Neumüller, Alexandra Lang, Sibylle Madlener, Anna Laemmerer, Katharina Bruckner, Carola N. Jaunecker, Bernhard Robl, Romina Walter, Julia Schueler, Andreas Peyrl, Amedeo A. Azizi, Christian Dorfer, Karl Roessler, Sabine Spiegl-Kreinecker, Christine Haberler, Aniello Federico, Apurva Gopisetty, Gudrun Schleiermacher, Stefan M. Pfister, Birgit Geoerger, Louis Chesler, Gilles Vassal, Marcel Kool, Leonhard Müllauer, Carl Koschmann, Walter Berger, Johannes Gojo, Daniela Lötsch

**Affiliations:** 1 Department of Pediatrics and Adolescent Medicine, Medical University of Vienna, Vienna, Austria; 2 Comprehensive Center for Pediatrics, General Hospital Vienna, Vienna, Austria; 3 Comprehensive Cancer Center, General Hospital Vienna, Vienna, Austria; 4 Department of Neurosurgery, Medical University of Vienna, Waehringer Guertel 18-20, Vienna, 1090 Austria; 5 Center of Cancer Research, Medical University of Vienna, Vienna, Austria; 6 Department of Pediatrics, University of Michigan Medical School, Ann Arbor, Michigan USA; 7 Charles River Laboratories Germany GmbH, Freiburg, Germany; 8 Department of Neurosurgery, Johannes Kepler University, Kepler University Hospital Linz, Linz, Austria; 9 Clinical Research Institute for Neurosciences, Johannes Kepler University , Linz, Austria; 10 Department of Neurology, Division of Neuropathology and Neurochemistry, Medical University of Vienna, Vienna, Austria; 11 Hopp Children’s Cancer Center Heidelberg (KiTZ), Heidelberg, Germany; 12 German Cancer Research Center (DKFZ) and German Cancer Consortium (DKTK), Heidelberg, Germany; 13 National Center for Tumor Diseases (NCT), Heidelberg, Germany; 14 Children’s Oncology Research Unit, Research Center, PSL Research University, Institut Curie, Paris, France; 15 SIRIC Laboratory of Translational Research in Pediatric Oncology, Translational Research Department, Research Center, PSL Research University, Paris, France; 16 Department of Pediatric Oncology, Hematology & Immunology, Heidelberg University Hospital, Heidelberg, Germany; 17 Université Paris-Saclay, Inserm U1360, Gustave Roussy, Villejuif, France; 18 Department of Pediatric and Adolescent Oncology, Gustave Roussy Cancer Campus, Université Paris-Saclay, Villejuif, France; 19 Division of Clinical Studies, The Institute of Cancer Research, London, UK; 20 Princess Máxima Center for Pediatric Oncology, Utrecht, The Netherlands; 21 Department of Pathology, Medical University of Vienna, Vienna, Austria

**Keywords:** Pathogenic *BRAF* mutation, *CDKN2A/B* loss, Pediatric high-grade glioma, CDK4/6 inhibitor, Abemaciclib, Mesenchymal

## Abstract

**Background:**

*BRAF*-altered pediatric high-grade gliomas (pHGG) harbor a dismal prognosis. Although targeted therapy with BRAF and MEK inhibitors provides initial benefit, resistance emerges rapidly in clinical practice indicating an urgent need for improved therapeutic strategies. As *BRAF* mutations frequently co-occur with homozygous *CDKN2A/B* loss, we investigated CDK4/6 inhibition as a rational therapeutic strategy.

**Methods:**

Using *BRAF-*mutant cell and human-to-organoid transplant (HOT) models, we assessed the impact of CDK4/6 and MEK inhibitors as mono- or combination therapies on viability, apoptosis, senescence, and molecular signaling. Activation of signaling pathways in primary and recurrent matched samples pre- and post-combined MEK and BRAF treatment was examined by RNA sequencing. In vivo efficacy was evaluated in orthotopic and subcutaneous patient-derived xenograft (PDX) models, as well as in one clinical case.

**Results:**

*BRAF*-mutant, *CDKN2A/B*-deficient pHGGs displayed strong sensitivity to the CDK4/6 inhibitor abemaciclib in addition to the MEK inhibitor trametinib. These tumors were particularly vulnerable to combined abemaciclib and trametinib treatment, which induced senescence and apoptosis, and uniquely suppressed mTOR activity. Both HOT and PDX models exhibited tumor regression, prolonged survival and sustained response even after therapy discontinuation. These effects were accompanied by a decreased mesenchymal-like cellular phenotype, as indicated by lower CD44 expression and a shift toward a more rounded cell morphology. Interestingly, trametinib- and dabrafenib post-treatment samples exhibited further increase in CD44 levels, along with upregulation of PI3K and hypoxia signaling, indicating therapy-associated reinforcement of mesenchymal transition.

The combination of trametinib and ribociclib was translated into clinical application, by showing good response of a patient with BRAF-altered and therapy-refractory pHGG.

**Conclusion:**

Our study demonstrated enhanced and prolonged effects of combined CDK4/6 and MEK inhibition in *BRAF*/*CDKN2A*-co-altered recurrent pHGG. We further provide evidence that the aggressive mesenchymal cell compartment is particularly targeted by this treatment combination, warranting further preclinical and clinical investigation.

**Graphical Abstract:**

**Supplementary Information:**

The online version contains supplementary material available at 10.1186/s13046-026-03725-3.

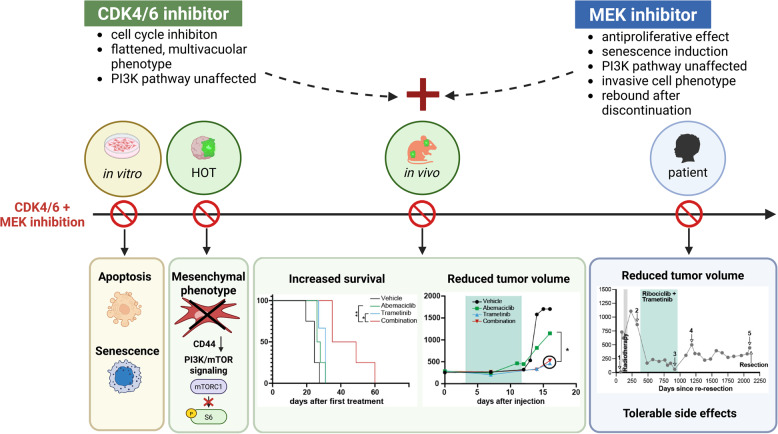

## Background

Pediatric high-grade gliomas (pHGG) have a dismal prognosis and account for approximately 10% of childhood central nervous system (CNS) tumors [1]. First-line therapeutic gold standard still consists of maximal safe resection or biopsy depending on the tumor location, followed by local radiotherapy. Upon tumor recurrence, no effective treatment is available and different options including re-irradiation, chemotherapy, immunotherapy and tyrosine kinase inhibitors are applied [1]. High-throughput technologies enabled deeper insights into molecular drivers and opened the field for precision medicine and targeted therapy. *BRAFV600E* mutations occur in approximately 5–15% of pHGG [2] and initially respond well to the oral targeted therapeutics dabrafenib alone [3] and in combination with trametinib, targeting BRAF*V600E* and MEK, respectively [4].Both inhibitors are approved as a second-line approach in pHGG patients older than 6 years without other satisfactory therapeutic options [4]. *BRAF*-mutant (BRAFmut) HGGs per se demonstrate phenotypic heterogeneity and cell plasticity [5] contributing to the observed intrinsic resistance [5] Additionally, BRAFmut HGG cells may escape BRAF/MEK inhibition, leading to the development of resistance mechanisms after therapy discontinuation, tumor rebound or progression [6, 7].

Besides *BRAF* being the oncogenic driver in these cancers, second hits such as homozygous *CDKN2A/B* loss and *TERT* promoter (*pTERT*) mutations are required to allow infinite proliferation in tumor cells and prevent induction of senescence [8, 9]. Thus, homozygous *CDKN2A*/*B* loss co-occurs in approximately 60% of *BRAF*-mutant pHGG [10] and is considered a marker of aggressiveness [11]. It leads to hampered functionality of the cyclin-dependent kinase 4/6- dependent (CDK4/6) G1-/S phase checkpoint and subsequently to uncontrolled cell proliferation. Currently, the CDK4/6 inhibitors palbociclib, ribociclib, and abemaciclib are FDA- and EMA-approved in other cancer indications [12], including hormone receptor (HR)-positive, human epidermal growth factor receptor 2 (HER2)-negative advanced or metastatic breast cancer, in combination with an aromatase inhibitor or for patients with prior endocrine therapy with fulvestrant [13]. Although abemaciclib was specifically designed to cross the blood-brain barrier (BBB) [14], studies demonstrated that ribociclib also exhibits good BBB penetrance in pHGG [15, 16] and in adult patients with recurrent glioblastoma [17].

Given the rationale that *BRAF* mutations frequently co-occur with *CDKN2A/B* loss in pHGGs, we hypothesize that combined MEK and CDK4/6 inhibition exhibits enhanced anti-tumor activity, also in therapy-refractory cases. Thus, we investigated the impact of CDK4/6 and MEK inhibition on cellular and molecular characteristics in matched primary and therapy-refractory preclinical BRAFmut HGG models with *CDKN2A/B* loss, including patient-derived cell and xenograft models as well as human-to-organoid transplants. Finally, our study demonstrates the first clinical applicability of combined treatment with MEK and CDK4/6 inhibitors in a pediatric *BRAF*mut HGG.

## Materials and methods

### Cell lines and cell culture

Two previously published pediatric patient-derived cell models from the Medical University of Vienna, VBT92 (anaplastic pleomorphic xanthoastrocytoma) and VBT125 (recurrent gliosarcoma, pretreated with trametinib/dabrafenib), were cultured in RPMI-1640 with 10% FCS [9]. Adult glioblastoma models AM38 (Japanese Collection of Research Bioresources Cell Bank) cultured in Eagle’s minimal essential medium with 20% FCS, and DBTRG-05MG (Deutsche Sammlung von Mikroorganismen und Zellkulturen GmbH, Braunschweig, Germany), along with patient-derived BTL53 and BTL1529 (Kepler University Hospital in Linz), were cultured in RPMI-1640 (7–10% FCS). All media contained 2% L-glutamine and 0.2% Normocin (Sigma-Aldrich, St. Louis, Missouri, USA). Cells were maintained at 37 °C with 5% CO₂ and regularly checked for *Mycoplasma* contamination. Cell line characteristics are provided in Table 1. Molecular alterations were analyzed with different methods, including Whole Exome Sequencing for VBT92 and VBT125, or targeted next-generation sequencing with the Ion AmpliSeq™ Cancer Hotspot Panel v2 for BTL53 and BTL1259. Biological information from commercially available cell models was derived from the COSMIC database [18].

**Table 1:** Characteristics of patient-derived cell models in our cohort

Cell line	Age (years)	Sex	Histology	BRAF	CDKN2A expression	Additional alterations
DBTRG-05MG	59	F	GBM	V600E	negative	pTERT, POT1
AM38	36	M	GBM	V600E	negative	pTERT, ALK
VBT92	12	F	aPXA	V600E	negative	pTERT
VBT125	13	F	gliosarcoma	V600E	negative	pTERT
BTL53	45	M	GBM	WT	positive	PTEN, RB1
BTL1529	30	F	GBM	WT	negative	pTERT, PTEN, TP53

*F* Female, *M* Male, *GBM* Glioblastoma, *aPXA* Anaplastic pleomorphic xanthoastrocytoma, *WT* Wildtype

### Drugs

All drugs used in this study were purchased from Selleck Chemicals (Houston, TX, USA). For experimental use, aliquots with a concentration of 10 mM were dissolved in dimethyl sulfoxide (DMSO) and stored at -20 °C.

### Cell viability (ATP) assay

Cells were seeded in triplicates (2.5 × 10⁴ cells/mL) in black 96-well plates (Thermo Fisher Scientific Inc., Waltham, Massachusetts, USA) and allowed to attach for 24 h. Abemaciclib, palbociclib, ribociclib, trametinib, afatinib, and avapritinib were added in 100 µL growth medium with 10% FCS at concentrations ranging from 0 to 10 µM and incubated for 72 h. DMSO was added to the control wells at a concentration mimicking the highest drug concentration. The proportion of viable cells was determined by ATP assay following the manufacturer’s recommendations (CellTiter-Glo^®^ luminescent cell viability assay, Promega, Madison, WI, USA). Luminescence was measured at 1000 nm using the Tecan infinite 200Pro (Zurich, Switzerland). Raw data were analyzed using GraphPad Prism 8.0 software (GraphPad Software Inc., La Jolla, CA, USA). Results are given as mean +/− SD and were normalized to untreated control cells. Cytotoxicity was expressed as IC_50_-values calculated from full dose–response curves (drug concentrations inducing a 50% reduction of cell number in comparison to the untreated control cells).

### Spheroid assay

For investigation of long-term toxicity effects, cells (5–10 × 10^4^ cells per well) were seeded in 100 µL of a 1:1 mixture of Neurobasal™ Medium and Dulbecco’s Modified Eagle Medium/Nutrient Mixture F-12 (1:1) (Thermo Fisher Scientific Inc.). The medium was supplemented with GlutaMAX™-I, B-27^®^ Supplement without vitamin A, N-2 Supplement, human EGF, human FGF-basic (all purchased from: Thermo Fisher Scientific Inc.), as well as MEM Non-essential Amino Acid Solution and human insulin solution (Sigma-Aldrich) and Heparin Sodium (STEMCELL, Vancouver, British Columbia, Canada). Cells were plated in triplicates in 96-well U-bottom plates. Following 24 h of recovery, 1µM of the CDK4/6 inhibitor abemaciclib or the MEK inhibitor trametinib were applied either as monotherapy or as combination therapy with both drugs. Treatment was reapplied every 72 h. The non-invasive live-cell imaging system Incucyte^®^ S3 (Sartorius AG, Göttingen, Germany ) was used to monitor sphere size for approximately 21 days (every 12 h). For quantification, images were preprocessed in ImageJ by converting them to 8-bit grayscale, followed by contrast adjustment to enhance the visibility of object boundaries. A binary mask of the object was generated by applying a global intensity threshold. Subsequently, the binary object was converted into a region of interest (ROI) and geometric parameters, including area, were obtained using the measurement functions.

### Cell cycle analysis

The effect of CDK4/6 and/or MEK inhibition on cell cycle distribution in our cell models was assessed by the Incucyte^®^ Cell Cycle Green/Red Lentivirus Reagent. To label cells of interest for cell cycle progression analysis, a lentiviral transduction system was used. After overnight incubation of the seeded cells a multiplicity of infection (MOI) of two per cell of the lentiviral reagent was added to the cells as well as 10 µg/mL Polybrene to increase the success of transduction. Cells were monitored via Incucyte^®^ Live-Cell Imaging System, and puromycin (0.5–1 µg/mL) selection was started when a fluorescence signal was detected. Cells were frozen or used for experiments when a homogeneously labeled cell population was established. Cells remained under puromycin selection during cell maintenance. Transfected cells were seeded in 500 µL of their selection medium (5 × 10^3^ cells/well) in a 24-well plate. After overnight incubation, a double block with 2mM thymidine (incubation for 16 h in medium with 2mM thymidine, followed by an 8-hour release phase) was performed to synchronize all cells into the same phase of the cell cycle. Treatment was initiated three hours after the second release with either 500 nM abemaciclib, palbociclib, trametinib, or a combined treatment of 250 nM abemaciclib or palbociclib and trametinib. Cells were imaged in the Incucyte^®^ Live-Cell imaging system for 4 days and cell cycle distribution was analyzed with the “cell-by-cell analysis” tool and analysis definitions based on the respective cell type were applied using the Incucyte^®^ analysis program.

### Apoptosis and senescence detection

Cells (1 × 10^4^/well) were seeded in 24-well plates and treated the next day with abemaciclib (2.5 µM), trametinib (1 µM), or a combination. Apoptosis was assessed either by propidium iodide (PI) staining or by using a caspase-3/7 assay (Incucyte^®^ Caspase-3/7 Green Dye) 24 h post-treatment. Senescence was evaluated by β-galactosidase staining (CST kit #9860) according to the manufacturer’s instructions. Regarding propidium iodide (PI) staining, PI was added to the cells, followed by an incubation for 1–2 h. The plates were subsequently imaged using the Incucyte live-cell analysis system to quantify apoptotic cells. Cells were washed to remove PI, and subsequently, induction of senescence was investigated by performing a β-galactosidase staining (Senescence β-Galactosidase Staining Kit #9860, Cell Signaling Technology). On the following day, the cells were observed under a microscope and images were taken. For quantification, cells were manually counted using ImageJ, and the percentage of senescent and apoptotic cells was determined by calculating the proportion of blue-stained cells relative to the total number of cells. In parallel, induction of apoptosis was further assessed by a caspase-3/7 assay. Following mono-or combined treatment with CDK4/6 and MEK inhibitors, cells were stained with the Incucyte^®^ Caspase-3/7 Green Dye and quantified using the Incucyte^®^ live-cell imaging system. The assay was performed according to the manufacturer’s instructions. Cells were treated with 2.5 µM abemaciclib, 1 µM trametinib, or a combination of both inhibitors and imaged 24 h post-treatment.

### Protein isolation and western blotting

**Fig. 1:**
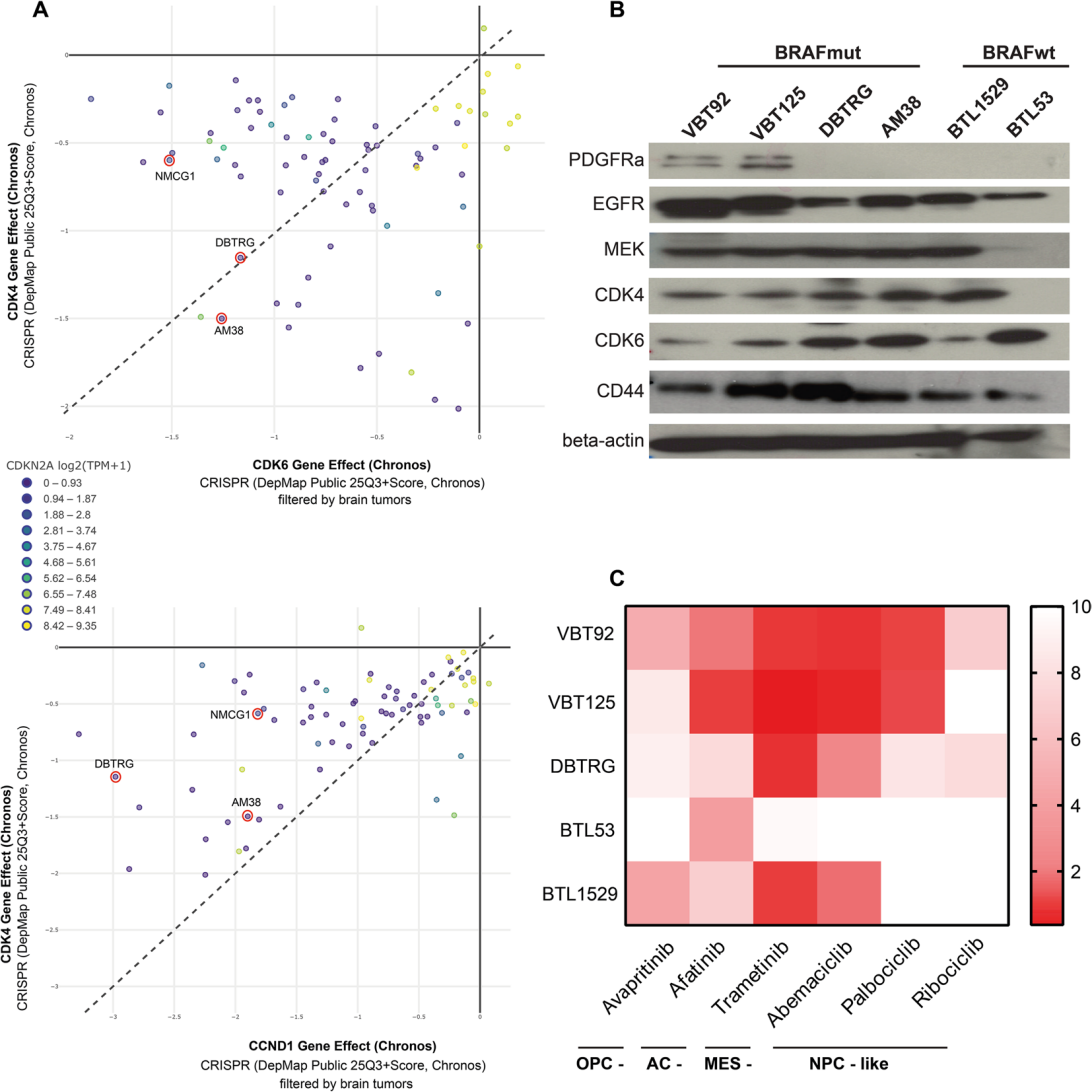
Molecular alterations and effects of tyrosine kinase inhibitors in *BRAF*-mutant high-grade glioma models. **A** The Depmap portal was used to investigate the dependency on *CDK4* and *CDK6* gene expression of different HGG cell models with *BRAF* mutation and different levels of *CDKN2A/B* expression (indicated by names; upper panel), as well as the dependency on *CDK4* and *CCND1* gene expression (lower panel). **B** Western blot analyses of frequent cellular subtype-specific alterations (PDGFRA for OPC-like, EGFR for AC-like, CDK4 for NPC-like, CD44/MAPK for MES-like) in HGG were performed in untreated cell models without and with *BRAF* alterations to further validate the findings from the CRISPR screens. Beta-actin was used as a loading control. **C** Preclinical cytotoxicity assessment of clinically approved tyrosine kinase inhibitors related to the cellular subtype-specific alterations (indicated below the drug names) in our patient-derived cell models by ATP assay after 72 hours of treatment with the indicated inhibitors ranging from 0-10µM concentration. The depicted values are the respective IC_50_ (red 0 and white exceeding 10µM) for the different inhibitors. OPC: oligodendrocyte progenitor cell; NPC: neural progenitor cell; AC: astrocyte; MES: mesenchymal

Western blotting was performed as previously described [19]. Basal protein levels of commonly dysregulated targets in high-grade glioma (Fig. 1B) were assessed in untreated cells under standard culture conditions. For the assessment of intracellular signaling, high-grade glioma cell models were seeded in six-well plates (two wells per condition) and treated for 72 h with either 500 nM CDK4/6 inhibitors (abemaciclib, palbociclib, ribociclib), the MEK inhibitor trametinib, or a combination of both. After 72 h, protein extraction and Western blot analysis were carried out according to published protocols. Primary antibodies (Supplemental Table S1) were diluted 1:1000 in 3% BSA in Tris-buffered saline containing 0.1% Tween-20 [20].

### Human-to-organoid-transplants

Cerebral organoids were established from H9 (female) human embryonic stem cells (hESCs; WiCell, Madison, Wisconsin) following the Lancaster protocol with minor adaptations [21]. These experiments were performed with two different batches of H9 hESCs. After embryoid body formation, germ layer induction, and neural induction, the organoids were embedded in Matrigel and placed in a 12-well plate followed by a switch to neural maturation medium on day 14. Subsequently, the organoids were maintained in a 6-well plate on an orbital shaker for further experiments. On day 90 of cerebral organoid growth, GFP-labeled VBT125 pediatric brain tumor cells were incorporated by injection of 2000 cells using a microinjector (MICRO-ePUMP). Treatment with 0.5 µM of each compound (abemaciclib and trametinib) as mono- or combination therapy was administered from day 110 to 122, followed by a post-treatment observation period of 12 days using the live-cell imaging system Incucyte^®^. Twenty-four hours before fixation, organoids were treated again. On day 134, one organoid of each treatment group from one batch was fixed and processed further as described in the immunohistochemistry section. Intensity of fluorescence imaging (GFP) representing tumor cell growth within organoids was determined using ImageJ. Images were separated into individual color channels using the *Image > Color > Split Channels* function. Regions of interest were outlined, and the integrated density and area were measured (*Analyze > Measure*). The normalized green intensity was calculated as the ratio of integrated density to the area (Normalized Green Intensity = Integrated Density / Area).

### Orthotopic mouse model

Orthotopic animal experiments were conducted in an AAALAC-accredited facility under permit G23/036 (Regierungspräsidium Freiburg, Germany). VBT125 tumor cells, transduced with iRFP-713 via lentivirus (EF1A promoter), were implanted intracranially into female NOD scid gamma (NSG) mice. By using a stereotactic frame, 4 µL of tumor suspension (1 × 10⁵ cells) was injected per mouse at 1 mm posterior, 2 mm right of bregma, and 3 mm depth. Each treatment group included four mice, with therapy starting two days post-implantation. The vehicle control received 5 mL/kg of Vehicle X (10% Cremophor-EL, 10% PEG400, 80% WFI), the trametinib group received 5 mL/kg corresponding to 0.3 mg/kg/day trametinib dissolved in Vehicle X, the abemaciclib group received 5 mL/kg corresponding to 50 mg/kg/day abemaciclib dissolved in Vehicle Y (1% hydroxyethyl cellulose; HEC in 25mM Na-phosphate buffer, pH 2) and the abemaciclib/trametinib group received 5 mL corresponding to 50 mg/kg/day abemaciclib and 0.3 mg/kg/day trametinib dissolved in Vehicle X daily via oral gavage. Imaging was performed at weekly intervals and if possible before termination. Tumor load was determined by in vivo imaging (IVI), utilizing the model’s iRFP tag, performed on a LI-COR Pearl^®^ Trilogy imaging device. Based on the specifications of the imaging device, in vivo imaging was performed at 685 nm excitation and 720 nm emission wavelength. Body weights were measured three times per week throughout the study. In addition to body weights and in vivo imaging as euthanasia criteria, mice were observed for clinical activity. Animals with a score = 3 for more than two days (i.e. three consecutive examinations) or mice with a score **<** 3 for one day were euthanized. Due to the aggressive nature of the VBT125 model, treatment lasted 14 days, and remaining animals were sacrificed by cervical dislocation on day 16. Mouse brains were collected as FFPE samples.

### Subcutaneous mouse model

Subcutaneous animal experiments were performed based on authorization by the Ethics Committee of the Medical University of Vienna and Austrian Ministry for Science and Technology (GZ 2023 − 0.122.324 and GZ 2024 − 0.901.338) and followed the guidelines of the Federation of Laboratory Animal Science Associations (FELASA) as well as the ARRIVE guidelines for animal care and protection. Planning of the experiments considered in all cases the strategies to replace, reduce, and refine (“3R”). Endpoints included excessive tumor burden (> 1.5 cm diameter), ulceration or animal weight loss (> 15% of pre-treatment weight), in accordance with the guidelines for the welfare and use of animals in cancer research [22]. Subcutaneous tumor growth was initiated by injecting 1 × 10^6^ VBT125 cells into the right flank of 6-8-week-old SCID/BALBc male mice (Harlan Winkelman, Borchen, Germany). Each experimental group contained four mice. Body weight and tumor size were determined three times per week using a Vernier caliper [19]. Oral gavage treatment was started when tumors were detectable, three days after subcutaneous injection. Abemaciclib (50 mg/kg/day) was dissolved in the respective amount of 0.5% hydroxypropylcellulose (HPC) in 25mM sodium phosphate buffer at pH2 (= Vehicle I). Trametinib (0.3 mg/kg/day) was dissolved in the respective amount of 0.5% hydroxypropylmethylcellulose (HPMC) and 0.2% Tween in ddH2O (= Vehicle II). Each drug was administered orally in a final volume of 100 µl/20 g body weight (BW) together with 100 µl of the complementary vehicle. The solvent group received 100 µl/20 g BW of Vehicle I and 100 µl/20 g BW of Vehicle II. Treatment was administered for 10 days, followed by an observational period without treatment.

### Immunohistochemistry

Subcutaneous, orthotopic, and patient tissues were fixed in 4% formaldehyde overnight; organoids in 4% paraformaldehyde for 1 h on a roller shaker. After fixation, samples were dehydrated and paraffin-embedded. Consecutive tissue sections (2 μm for subcutaneous/orthotopic, 1.5 μm for organoids) were deparaffinized, rehydrated, and treated with 0.3% H₂O₂ to block endogenous peroxidases. Antigen retrieval was performed using heat and pH-specific Target Retrieval Solutions (Dako K8005/K8002 Agilent, Santa Clara, CA, USA). Slides were blocked with 5% horse serum (Sigma, H1270) and incubated overnight at 4 °C with primary antibodies (see Supplemental Table S2) in PBS + 1% BSA + 0.1% Tween-20. The Agilent EnVision™ HRP Kit (K5007) was used for detection, with Mayer’s hemalaun for counterstaining. Stained slides were digitized using a high-resolution slide scanner at 20x magnification (Evident Scientific VS200 for organoids and 3DHistech Pannoramic SCAN II for patient-derived xenograft tissue). The intensity of staining was analyzed in ImageJ via H-DAB color deconvolution followed by inverse grayscale conversion, with lower pixel values indicating stronger staining. Regions of interest (ROI) were defined within each sample according to GFP-positive areas. The integrated intensity and area within each ROI were extracted (*Analyze > Measure*), and mean staining intensity was calculated as the integrated density normalized to the ROI area. Background signals were estimated from unstained areas and subtracted from the measurements. To determine cellular shapes (roundness values;1 = perfect circle; <1 more elongated shape) the function “Analyze Particles” was used.

### Clinical samples and patient data

Tumor tissues for analyses and establishment of patient-derived cell models VBT92 (VBT92; primary tumor; pre-treatment) and VBT125 (VBT125 ; recurrent tumor; post-treatment) were derived from a patient treated at the Department of Pediatrics and Adolescent Medicine at the Medical University of Vienna. The recurrent tumor tissue was collected after eight months of trametinib and dabrafenib treatment. In addition, tissue from the MUV_02 patient (compare Fig. 4D), a pediatric patient with an H3K27-altered thalamic diffuse midline glioma with a *BRAFV600E* mutation, was investigated [23]. The primary tumor tissue was taken before initiation of treatment, and the recurrent tumor tissue from the autopsy after treatment with trametinib and dabrafenib for seven months. Both patients were treated with resection, radiotherapy as well as trametinib and dabrafenib before progression. Histopathological diagnoses were assessed by experienced neuropathologists according to the 2021 WHO classification. Clinical histories and characteristics were obtained from patient charts available at the respective hospitals. The study was approved by the local institutional review board of the Medical University of Vienna (EK Nr. 1244/2016). Informed consent was obtained from all patients and/or their legal representatives.

The clinical case was treated at the Department of Pediatrics at the University of Michigan’s Mott Children’s Hospital. Molecular testing was completed through the IRB-approved Pediatric MiOncoSeq [24] study for next-generation DNA and RNA sequencing of tumor and germline samples using previously established methods (HUM00056496). Clinical information was acquired by retrospective chart review, and imaging analysis was completed using manual segmentation, IRB non-regulated (HUM00273528).

#### Analyses of HGG samples within ITCC-P4

As members of the European project “IMI ITCC-P4”, our group has access to a cohort of comprehensively molecularly characterized brain tumor subtypes and matching patient-derived xenografts (PDX). Thus, we analyzed RNA sequencing data from 21 HGG PDX samples (with confirmed diagnosis by DNA methylation) including three primary *BRAFV600E* mutant cases (HG0067 matching VBT92, HG0355 (GR-HGG-5) and HGG0356 (GR-HGG-9)) [25] available via the R2 ITCC-P4 PDX Data scope portal (https://r2platform.com/itcc-p4/*).* RNA sequencing of patient tumors and PDX models was performed on Illumina HiSeq instruments. Data were processed, including read alignment with STAR, gene expression quantification with FeatureCounts, and gene fusion detection with Arriba. The processed expression data were used for differential gene expression and gene ontology analyses.

#### Analyses of matched *BRAFV600E* HGG samples pre- and post-treatment

The bulk RNA-seq data (FFPE, paired-end reads 2 × 150) of patient 12, a 13-year-old boy with a *BRAFV600E*-mutant pHGG was downloaded for analysis under GEO accession number GSE287972 [26]. This includes two measurements, one pre-BRAFi+MEKi treatment and one post and were obtained as a gene-level count matrix [26, 27] (raw FASTQ files were not available). Only gene-level read counts (no raw FASTQ files) were available for our study. These counts were generated by Xing YL et al. [27] using STAR aligned to hg19 and featureCounts and used as provided.

Patient-derived cell lines VBT92 and VBT125 were sequenced in-house. Library preparation was performed with polyA selection module, and NEBNext Ultra II Directional RNA with UMI Adaptors. The libraries were then sequenced on NovaSeq 6000 SP flow cell with paired-end reads (2 × 101 bp). In-house FASTQ files were processed with the nf-core [28]/rnaseq pipeline [29] (v3.18.0, Nextflow v24.10.3) using fastp for trimming and STAR for alignment to GRCh38.p14 and Salmon for quantification.

We performed preprocessing of the raw sequencing data by running the nf-core pipeline rnaseq v3.18.0 via Nextflow (v24.10.3). GRCh38.p14 was used as a reference genome. The trimmer: ‘fastp’ was selected. The selected settings included: extra_fastp_args: ‘--length_required 20 --adapter_fasta adapter.fa --trim_front2 3 --cut_tail_window_size 4 --cut_tail_mean_quality 20 --trim_poly_g’. Adapter sequences of Illumina-TruSeq and polyA and polyT were included. For alignment these options were selected: extra_star_align_args: ‘--outFilterScoreMinOverLread 0.3 --outFilterMatchNminOverLread 0.3 --chimOutType WithinBAM’. All the other pipeline options were left to default.

Downstream analysis was performed in R (v4.4.3). Gene Set Variation Analysis (GSVA) was conducted on log2-transformed normalized expression data (GSVA R package v1.52.3, minSize = 10, maxSize = 500). Pathway activity scores were inferred using the PROGENy R package (v1.26.0). Unless otherwise specified, default parameters were applied [30].

### Statistical analysis

Statistical analysis was performed using GraphPad Prism 8.0 (GraphPad Software Inc., La Jolla, California, USA). All in vitro experiments were carried out independently at least three times. All data are expressed as mean +/− S.D. Statistical significance of differences was analyzed using unpaired Student’s t-test and one-way ANOVA with Bonferroni’s post hoc correction. p-values < 0.05 were considered statistically significant. Throughout the study, the following classification is used: *, *p* < 0.05; **, *p* < 0.01 ***, *p* < 0.001, ****, *p* < 0.0001.

### Data availability

RNA sequencing data from the R2 ITCC-P4 PDX Data Scope portal are available to the global scientific community upon access request. RNA sequencing data generated in-house from VBT92 and VBT125 will be uploaded to EGA upon publication.

## Results

### Distinct molecular alterations predict anti-cancer effects of CDK4/6 inhibitors in high-grade glioma

Guided by our hypothesis that both *BRAF* alterations and chromosomal loss of *CDKN2A/B* constitute targetable vulnerabilities in HGG, we initially investigated gene dependencies and their therapeutic potential using the DepMap portal (https://depmap.org/portal*).* In the group of malignant brain tumors, we observed a strong codependency of *CDK6* and *CDK4* (Fig. 1A, upper panel) as well as of *CDK4* and *CCND1* (Fig. 1A, lower panel) in *BRAF*-mutant HGG cell models with a concomitant *CDKN2A/B* loss. This subgroup comprises two publicly available HGG models, DBTRG05-MG and AM38, which we included in our study for further analyses, as well as an additional adult HGG cell line (NMCG1). To increase our preclinical cell panel, we further selected pHGG models, one primary (VBT92) and one matching recurrent (VBT125) case, from our lab biobank. Molecular and clinical characteristics of the investigated cell models are shown in Table 1. As intrinsic resistance to targeted therapies is frequently driven by intratumoral heterogeneity, we next assessed the expression of characteristic cell-state markers as published by Neftel et al. [5] in our *BRAF*-mutant (BRAFmut) and wild-type (BRAFwt) HGG models (Fig. 1B). Levels of EGFR, representing the astrocyte (AC)-like subtype, CDK4 and CDK6, markers for the neural-progenitor (NPC)-like subpopulation, and the mesenchymal (MES)-like proteins, CD44 and MEK, could be detected in almost all tested models, independent of *BRAF* status, although with varying levels. In particular, CD44 protein expression was distinctly higher in the BRAFmut cell models. When compared to the primary VBT92 pHGG case, CD44 was upregulated in the recurrent VBT125 cell model, established from the same lesion that progressed under combined targeted treatment with dabrafenib and trametinib. Similarly, PDGFRA, enriched in oligodendrocyte precursor (OPC)-like cells, was detected only in the two BRAFmut pHGG models, and its levels were further increased in the recurrent VBT125 cell line. By targeting each of the described cellular subtypes using respective tyrosine kinase inhibitors, we observed distinct cytotoxicity upon treatment with CDK4/6 inhibitors, particularly of abemaciclib and palbociclib, in models with *CDKN2A/B* loss and/or *BRAF* mutation (Fig. 1C). Inhibition of the MAPK/PI3K signaling cascade by trametinib (MEKi) resulted in strong anti-cancer responses in the investigated cell models, indicated by low IC_50_ values, while targeting PDGFRA (avapritinib, PDGFRA/KIT inhibitor) and EGFR (afatinib, pan-ErbB inhibitor) was not effective or less effective in the absence of the particular molecular driver alterations.

In conclusion, a subset of HGG with *BRAF* mutation and *CDKN2A/B* loss showed pronounced CD44 expression, a marker for the mesenchymal phenotype, paralleled by a strong dependency on CDK4/6 function. This was further underlined by increased sensitivity towards the CDK4/6 inhibitor abemaciclib and increased sensitivity to the already approved targeted inhibitor trametinib.

### Targeting *CDKN2A/B* loss and *BRAF* mutation results in synergistic induction of apoptosis and senescence as well as inhibition of PI3K-mTOR signaling

**Fig. 2:**
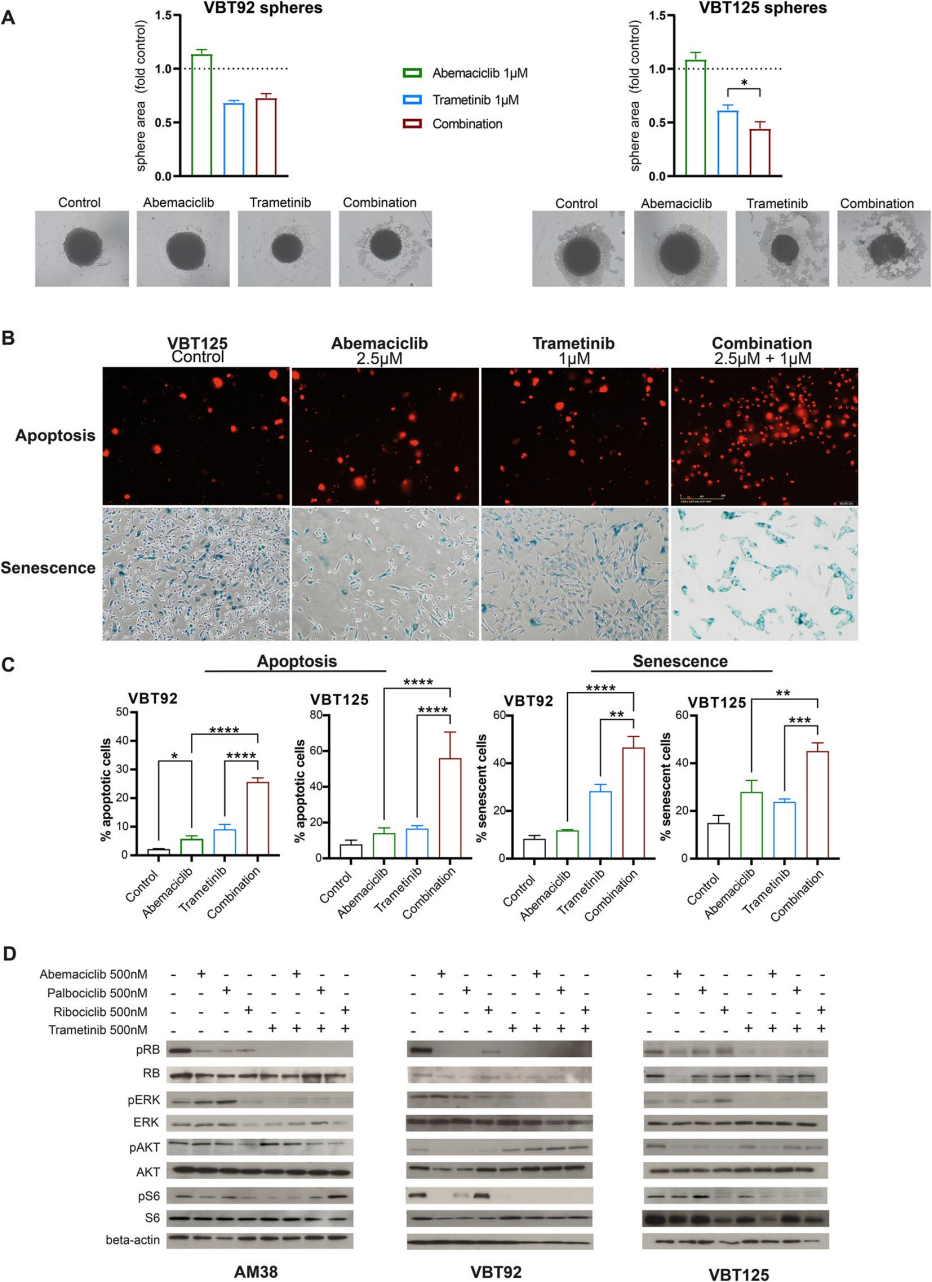
Combined CDK4/6-MEK inhibition triggers apoptosis and senescence, while reducing PI3K-mTOR signaling in *BRAF-*mutant HGG. **A** Spheroid assays. Cells (5–10 × 10⁴ per well) were seeded in 96-well U-bottom plates (100 µL medium; triplicates) and allowed to recover for 24 h. Abemaciclib and trametinib (1 µM each) were applied as monotherapies or in combination, with treatments re-applied every 72 h. Sphere growth was monitored for ~21 days at 12-h intervals using the Incucyte^®^ S3 Live-Cell Imaging System, and images were analyzed with ImageJ. One-way ANOVA was used to determine statistical significance and the data is presented as mean +/- SD. Representative images are shown after 10 days of treatment. **B** Representative images from the VBT125 model after 24 hours of treatment are depicted for apoptosis in the upper and senescence in the lower panel. **C** For quantification, cells were manually counted, and the percentage of senescent and apoptotic cells was determined by calculating the proportion of blue-stained cells relative to the total number of cells. Unpaired t-test was used to determine statistical significance, and the data is presented as mean +/- SD. **D** Cells were seeded and treated 24 hours later with 500 nM of the respective drugs and combinations for 72 hours. Cells were lysed, proteins harvested and Western blot analysis performed as described in the methods section. All primary antibodies that were used are listed in Supplemental Table S1. Statistical significance is denoted as follows: *p* ≤ 0.0001 = ****, *p* ≤ 0.001 = ***, *p* ≤ 0.01 = **, and *p* ≤ 0.05 = *

As the single agents abemaciclib and trametinib showed the best efficacy in our mono-therapeutic setting when applied to adherently grown pHGG cell models, we investigated the combinatorial approach of these two targeting compounds further. To better model the clinical situation, we next tested the efficacy of MEK and CDK4/6 inhibitors as mono- or combination therapies on tumor spheres. Treatment with trametinib for 12 days distinctly reduced the spheroid size of the BRAFmut VBT92 and VBT125 models (Fig. 2A). However, in the recurrent, therapy-refractory cell model VBT125, the combinatorial treatment was significantly more effective in suppressing spheroid growth and inducing sphere degradation. Besides spheroid growth, we investigated the effect of CDK4/6 and MEK inhibition on cell cycle distribution in our cell panel. In VBT92 and VBT125 cells, monotherapy with abemaciclib, palbociclib, or trametinib, as well as the combination of abemaciclib and trametinib, resulted in a G0/G1 cell cycle arrest after 12 and 24 h (Supplemental Fig. 1A). As the combinatorial approach with trametinib and abemaciclib exhibited distinct anti-tumor effects and resulted in spheroid degradation in our cell panel, we interrogated whether this was mediated by apoptosis. Combination of abemaciclib and trametinib displayed a significantly higher level of apoptosis induction when compared to monotherapy, as tested by PI staining and caspase-3/7 expression (Fig. 2B-C, Supplemental Fig. 1B-C). As we observed an induction of G0/G1 cell cycle arrest upon treatment with CDK4/6 and MEK inhibitors, we next studied whether this was triggered by cellular senescence. The combinatorial approach with abemaciclib and trametinib significantly increased senescence in the investigated pHGG cell models when compared to monotherapy alone (Fig. 2B-C; images are shown for VBT125 in Fig. 2B, and for VBT92 in Supplemental Fig. 1B). Interestingly, in the VBT125 model, derived from the progressive, pretreated lesion, CDK4/6 inhibition more effectively induced a senescent phenotype comparable to trametinib monotherapy. In contrast, in the corresponding treatment-naive cell model VBT92, senescence levels induced by abemaciclib were distinctly lower and not significantly different compared to the control.

In line with the induction of a G0/G1 cell cycle arrest, RB and ERK phosphorylation was completely blocked upon treatment with trametinib alone or in combination with the investigated CDK4/6 inhibitors (Fig. 2D). In contrast, the monotherapy with CDK4/6 inhibitors did not efficiently inhibit MAPK signaling activation. While CDK4/6 monotherapy slightly reduced AKT phosphorylation, neither trametinib alone, nor the combination with the CDK4/6 inhibitors blocked PI3K signaling in the investigated cell models. Interestingly, the combination of trametinib and abemaciclib distinctly decreased the levels of pS6, one major downstream effector of the mTOR pathway, in all tested BRAFmut cell models. This observation is of particular importance as trametinib monotherapy exerted only a minimal effect on S6 phosphorylation in the BRAF/MEK inhibitor-pretreated VBT125 model, suggesting a bypass of MAPK pathway inhibition via the mTOR signaling pathway.

Together, these findings indicate that concomitant *CDKN2A/B* loss and *BRAF * alteration high-grade glioma display enhanced vulnerability to a combinatorial approach of abemaciclib and trametinib, primarily conferred via a concurrent or consecutive induction of senescence and apoptosis triggered by a G0/G1 cell cycle arrest. Exclusively when inhibiting both cell-cycle regulation and MAPK signaling, the activity of S6, a marker of mTOR pathway activation, was potently reduced.

### Combined abemaciclib and trametinib treatment overcomes mesenchymal differentiation

**Fig. 3:**
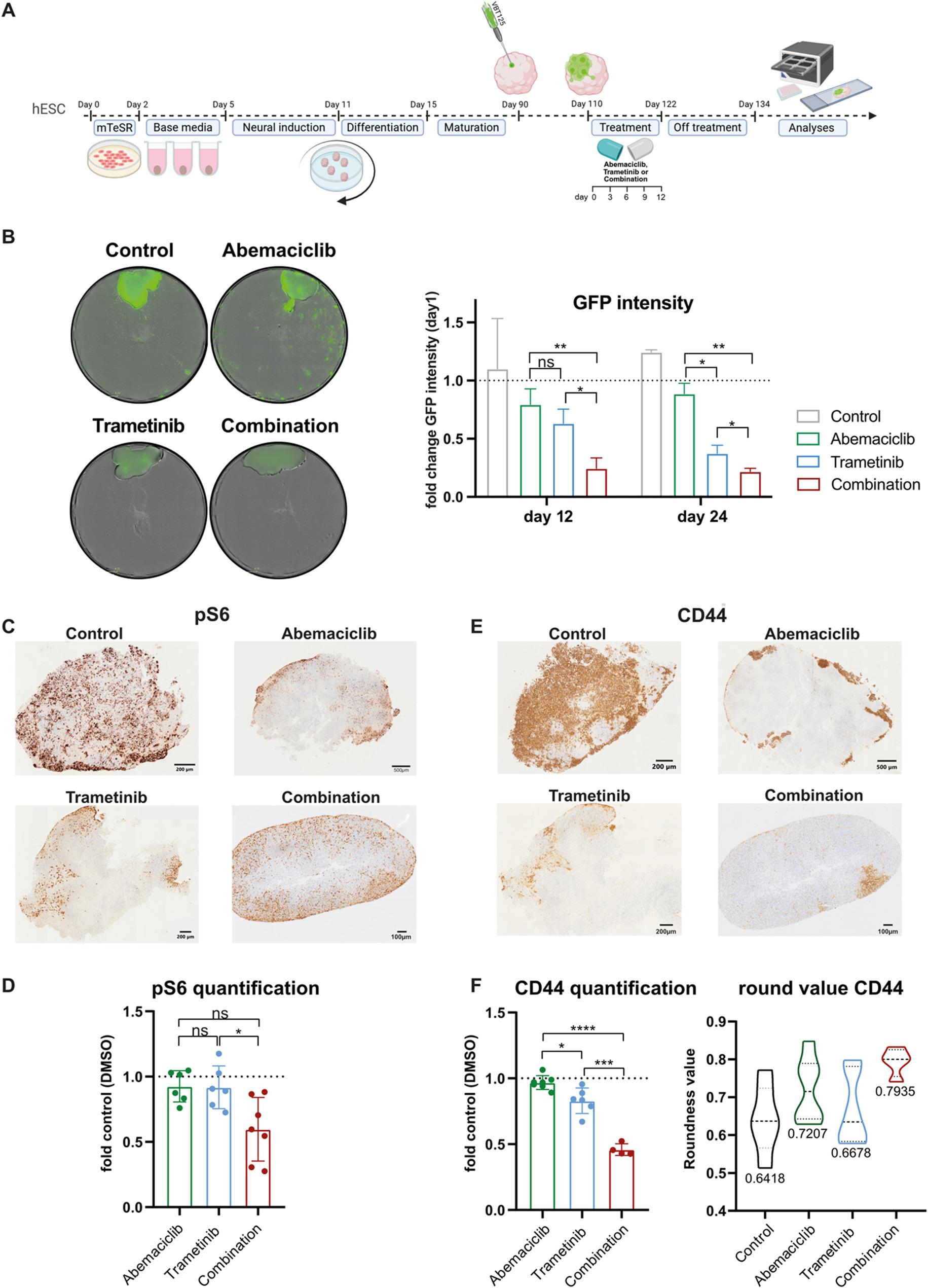
Preclinical activity of combined abemaciclib and trametinib in human-to-organoid-transplant models. **A** The HOT model of VBT125 was established as described in the methods section and the graphical overview of the timeline is depicted. **B** Fluorescence images of the organoids on day 21 are depicted (left panels). HOTs were treated with 0.5 µM of the respective drugs for 12 days. Fluorescence intensity representing tumor cell growth within organoids from three independent HOT batches at the indicated timepoints was determined using ImageJ. Normalized GFP intensity was calculated within ROIs as the ratio of integrated density to the area (Normalized Green Intensity = Integrated Density / Area) for the different timepoints (right panel). **C** Immunohistochemistry staining of pS6 was performed on the fixed and stained HOT sections and slides were scanned with a slide scanner for digitization. IHC images of pS6 positive cells in HOT models are depicted. **D** Mean staining intensity of pS6 quantified within regions of interest from two independent HOT batches was assessed using ImageJ and is depicted as fold control. **E** IHC images of CD44-positive cells in HOT models are depicted. **F** Mean staining intensity of CD44 quantified within regions of interest from two independent HOT batches was calculated using ImageJ and is depicted as fold control (left panel). Cellular shapes, expressed as roundness values (1=perfect circle; <1 more elongated shape), were quantified based on CD44-positive cells from HOTs of two independent batches using ImageJ (right panel). Scale bars in **C** and **E**: 100 or 200 µM as indicated. One-way ANOVA was used to determine statistical significance, and the data is presented as mean +/- SD. Statistical significance is denoted as follows: *p* ≤ 0.0001 = ****, *p* ≤ 0.001 = ***, *p* ≤ 0.01 = **, and *p* ≤ 0.05 = *.

Following up on these promising in vitro findings, we aimed to investigate the effects on more complex preclinical model systems. Thus, we generated human-to-organoid transplants (HOT) by fusing GFP-tagged VBT125 cells with cerebral organoids as depicted in Fig. 3A. These HOT models were treated with abemaciclib, trametinib, or the combination (0.5 µM each) for twelve days, followed by a period of twelve days without treatment, and GFP signal intensity was assessed. Untreated HOTs were almost completely infiltrated by VBT125 tumor cells, as shown by a representative image in Fig. 3B (left panel). In case of abemaciclib monotherapy, tumor cell growth was slightly decreased within the organoids, with signs of cell migration to the borders and outside of the HOT. Combinatorial inhibition of CDK4/6 and MEK demonstrated significantly reduced tumor cell survival when compared to trametinib monotherapy on day 12 (Fig. 3B, right panel). This combination also resulted in a significantly sustained treatment response, even following therapy withdrawal for 12 days (Fig. 3B, right panel and Supplemental Fig. 2A). To assess on-target effects and the impact on downstream signaling cascades, HOTs underwent IHC evaluation. In line with results from Western blot experiments, RB activation was reduced upon treatment with the investigated compounds as mono- or combination therapies (Supplemental Fig. 2A-B). Similarly, exposure to trametinib and to the combination with abemaciclib significantly reduced ERK phosphorylation, indicating on-target inhibition in HOT models (Supplemental Fig. 2C-D). Furthermore, confirming our Western blot results, the combined treatment approach significantly reduced pS6 levels when compared to mono-therapeutic trametinib (Fig. 3C-D). Although our data demonstrated anti-tumor activity of trametinib administered as monotherapy, the remaining tumor cells showed characteristics of infiltrative growth. This was indeed verified by evaluating protein expression of CD44, a marker of mesenchymal differentiation. CD44 protein levels were significantly and most prominently reduced in the combinatorial treatment arm, whereas only a weak inhibitory effect was observed following trametinib monotherapy, as assessed by IHC (Fig. 3E-F). Besides the expression of CD44, we further measured the shape of CD44-positive cells. VBT125 cells displayed enhanced roundness following combinatorial treatment, indicated by the highest roundness value (Fig. 3F, Supplemental Fig. 2E). In contrast, exposure to trametinib induced a more irregular, spindle-like morphology of tumor cells (Supplemental Fig. 2F), pointing toward an increased mesenchymal phenotype.

### Combined BRAF/MEK inhibitor treatment induces mesenchymal transition in *BRAF-altered* pHGG

**Fig. 4:**
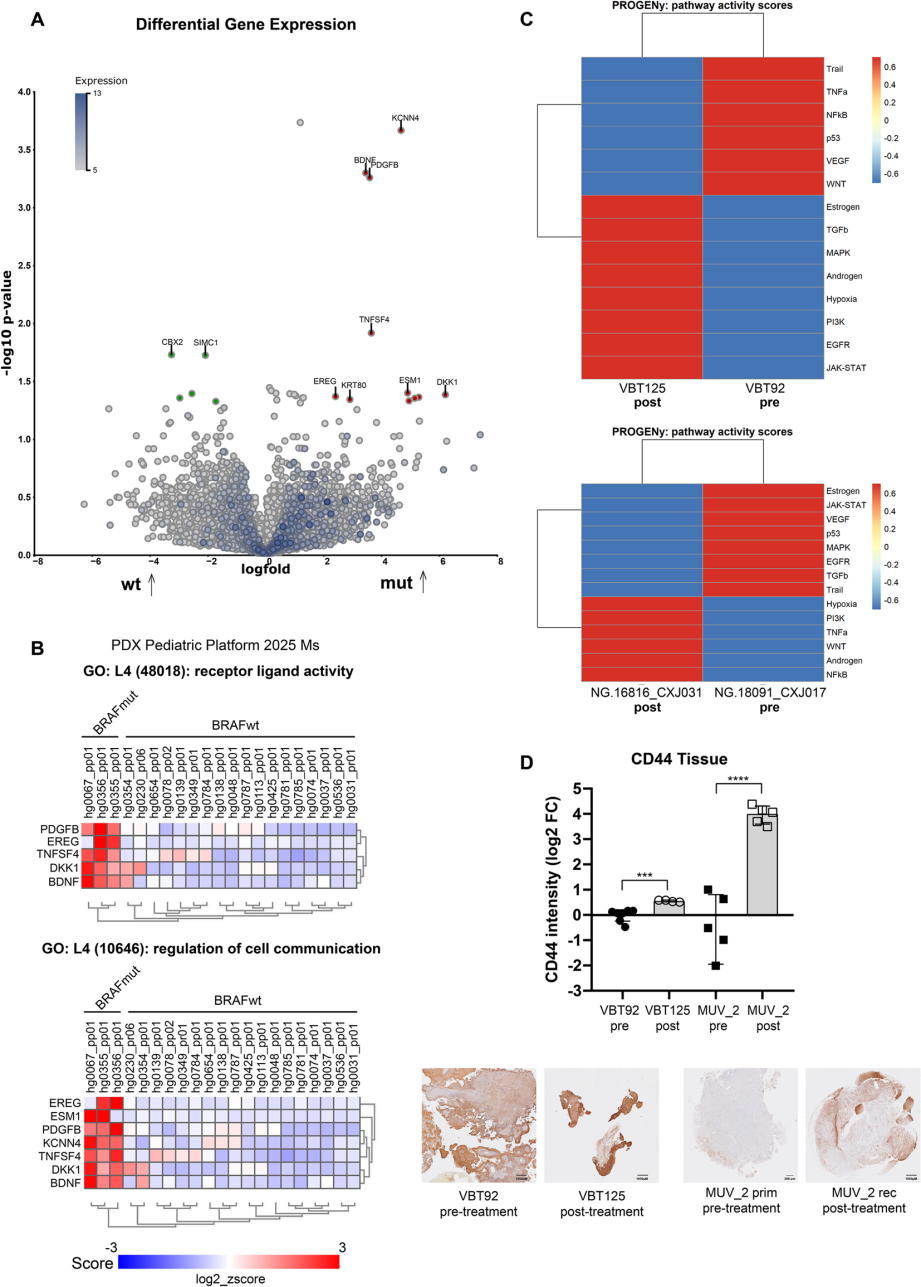
Activation of migratory signaling in *BRAF*-mutant HGG upon treatment with BRAF/MEK inhibitors. **A** Differentially expressed gene (DEG) analysis from RNA sequencing data of BRAFmut vs BRAFwt PDX models (n=21) extracted from the R2 platform (https://hgserver1.amc.nl) available via the ITCC-P4 database. Volcano plot depicts DEGs, highlighted as red or green dots, between the BRAFmut (*n*=3, primary tumors only), and BRAFwt (*n*=18) HGG cohort. **B** Selected significantly enriched Gene Ontology (GO) pathways derived from the DEGs in panel **A** are visualized as heatmaps between the indicated HGG groups. **C** Heatmaps depicting PROGENy-inferred pathways in two samples (VBT92 and VBT125 cell model and “patient 12”) [27] before and after treatment with combined BRAF/MEK inhibitors. Colors indicate pathway activation (red) or deactivation (blue). **D** Immunohistochemistry staining of CD44 in two BRAFmut patients before and after treatment with combined BRAF/MEK inhibitors. Intensity of CD44 quantified within regions of interest is compared for each case (VBT92/VBT125 and MUV_2) before and after therapy and is depicted as log2 fold change. IHC images of the investigated tissue section are shown. Scale bars in **D**: 200 µM or 1000 µM as indicated. Unpaired t-test was used to determine statistical significance denoted as follows: *p* ≤ 0.001 = ***, *p* ≤ 0.0001 = ****; pre=pre-treatment; post=post-treatment; prim=primary; rec=recurrent

To better interpret our findings, we continued to investigate transcriptional differences between BRAFmut versus BRAFwt pHGG. To this end, we analyzed RNA sequencing data of PDX models established from pHGG (*n* = 21) comprising BRAFwt and three primary BRAFmut samples (with confirmed *BRAFV600E* mutations), including the primary VBT92 case (HG0067). Differential gene expression analysis revealed 27 genes expressed at significantly different levels between the two groups (Fig. 4A, Supplemental Table S3). Among these genes, KCa3.1 (*KCNN4)*, encoding a calcium-activated potassium channel, and endothelial cell-specific molecule 1 (*ESM*1) were listed as top-ranked hits in the BRAFmut subgroup. Interestingly, both genes are associated with cancer cell migration and motility [31–33].

Differences in pathway activation were further investigated by Gene Ontology analysis, revealing, among others, activity of receptor signaling pathways and cell communication in BRAFmut as compared to BRAFwt pHGG tumors (Fig. 4B, Supplemental Table S4). Based on these findings, we were interested in how combined MEK and BRAF inhibition altered pathway activation in cell models (VBT92pre versus VBT125post) and in one additional matched case of a primary and therapy-refractory *BRAF*-mutant pHGG with *CDKN2A/B* loss [26, 27]. When applying PROGENy, a perturbation-based pathway model that estimates pathway activation using target-gene signatures, we discovered distinct activity patterns pre- and post-MEK/BRAF inhibitor treatment (Fig. 4C). In particular, the androgen, PI3K and hypoxia pathways were significantly upregulated in both post-treatment samples, suggesting their contribution to the development of resistance to the applied targeted therapeutics. Given that hypoxia can induce CD44 expression and thus supports tumor cell migration and invasion [34], we assessed the levels of CD44 in two *BRAF*mut pHGG cases (tissue matching VBT92, VBT125, and the MUV_02 case) before and after treatment failure with MEK/BRAF inhibitors. Indeed, CD44 levels were significantly increased after treatment with trametinib and dabrafenib in both tumor recurrences when compared to the respective treatment-naive primary tumor lesions (Fig. 4D), again highlighting the impact of BRAF and MEK inhibition on the mesenchymal phenotype in *BRAF-*mutant pHGG.

### Dual CDK4/6–MEK inhibition targets mTOR signaling and the mesenchymal phenotype in aggressive PDX models

**Fig. 5:**
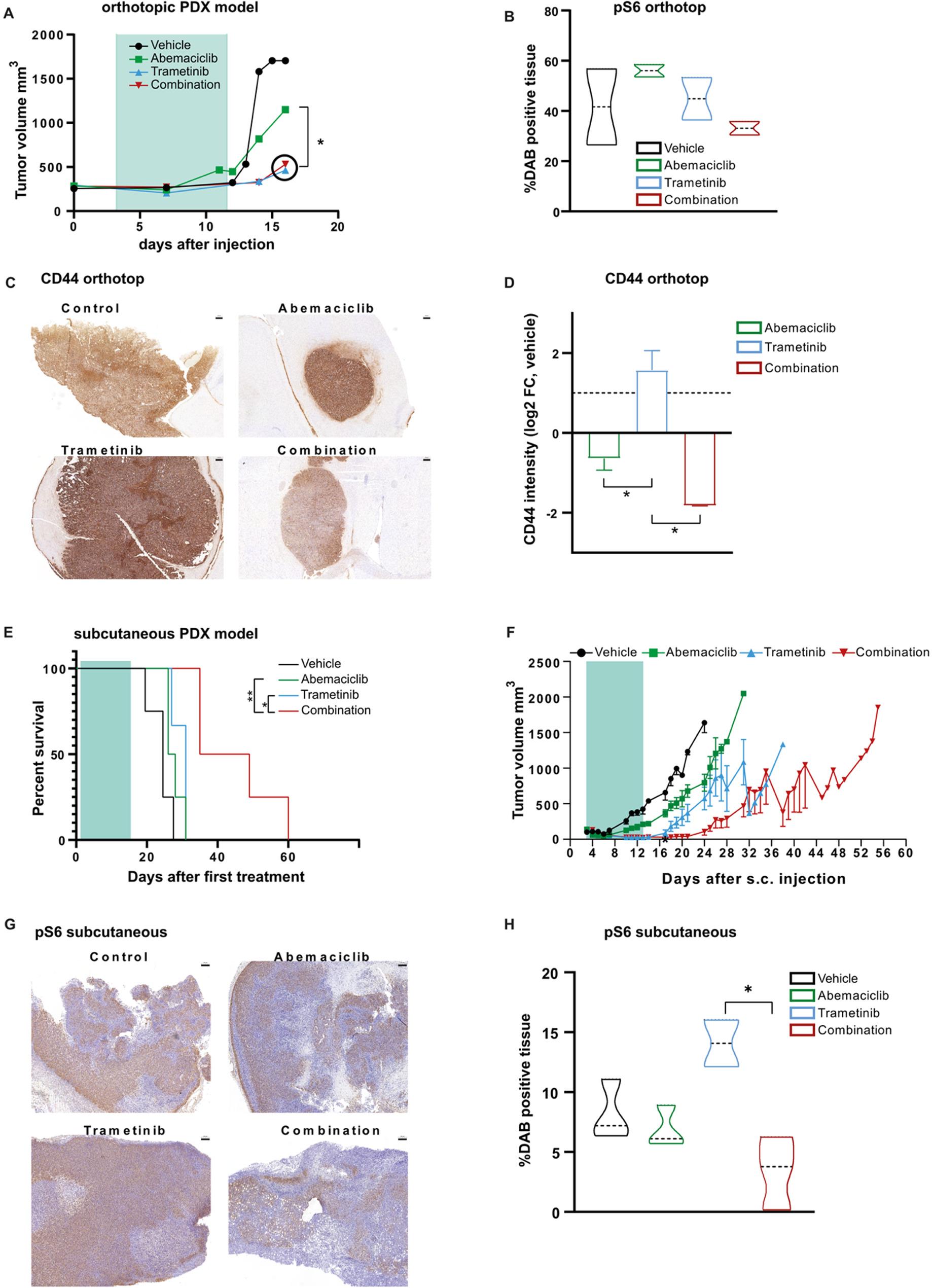
Preclinical activity of abemaciclib and trametinib in a patient-derived xenograft model. **A** VBT125 cells were orthotopically implanted in NSG mice (*n*=4 per group) and treatment was initiated three days after injection. Mean tumor volumes of the in vivo imaging (iRFP signal) in response to therapy are depicted as the last observation carried forward (LOCF) at the end point. **B** Assessment of IHC staining was performed with automated image analysis using HALO® Image Analysis Software to quantify the percentage of pS6-positive cells. **C** Images of CD44 protein expression in sections of orthotopic VBT125 xenografts treated as indicated. **D** CD44 expression was assessed using ImageJ. **E** VBT125 were subcutaneously injected in SCID/BALBc male mice and treatment with the depicted regimens (*n*=4 per group) was started three days after injection when subcutaneous tumors were palpable. Treatment was administered for ten days and overall survival and tumor volume was further assessed without treatment. Kaplan-Meier survival analysis was performed and unpaired t-test was used to determine statistical significance. **F** Tumor volume of the subcutaneous tumor was assessed three times per week. When the volume reached a size above 1500mm3, the animal was sacrificed. **G** Images of subcutaneous tumors collected at each individual endpoint after discontinuation of treatment were stained for pS6. **H** Immunohistochemistry of tumors was assessed with automated image analysis using HALO^®^ Image Analysis Software to quantify the percentage of pS6-positive cells. One-way ANOVA was used to determine statistical significance. Data are presented as mean +/- SD. Statistical significance is denoted as follows: *p* ≤ 0.01 = **, *p* ≤ 0.05 = *. Scale bars in G: 200 µM as indicated. The turquoise area depicts the treatment period.

Encouraged by these promising results, we next assessed the in vivo efficacy of combined abemaciclib and trametinib and examined their effects on the mesenchymal phenotype using a patient-derived xenograft model with orthotopically implanted VBT125 cells. Tumor volume was significantly decreased in the combination treatment cohort when compared to abemaciclib monotherapy and the control group (Fig. 5A). However, due to the rapid and highly aggressive tumor growth, no significant survival benefit was achieved upon combinatorial treatment (Supplemental Fig. 3A). Nevertheless, S6 phosphorylation was diminished (not significant) in the combined therapy arm as compared to single treatment, strengthening the impact on mTOR signaling cascade observed in cell and HOT models (Fig. 5B and Supplemental Fig. 3B). Analysis of orthotopic PDX tumors further confirmed the observations in HOT models, indicated by significantly reduced CD44 expression upon therapy with abemaciclib, or the combination, but not trametinib monotherapy (Fig. 5C-D). Interestingly, the cellular morphology remained unchanged upon treatment. In particular, combination treatment failed to reverse the spindle-shaped mesenchymal phenotype in the orthotopic xenograft model (Supplemental Fig. 3C and 3D), highlighting the importance of HOT models.

Due to the aggressive behavior of VBT125 in the orthotopic setting, we investigated the effects of CDK4/6 and MEK inhibitor combination on overall survival in the matching subcutaneous xenograft model. Subcutaneous VBT125 tumors were treated for ten days, followed by an observation period without treatment. Combined CDK4/6 and MEK inhibition resulted in significantly prolonged overall survival when compared to monotherapy with trametinib, abemaciclib, and the vehicle group (Fig. 5E). The combinatorial treatment arm showed sustained response after therapy discontinuation, depicted by distinctly reduced regrowth of subcutaneous tumors when compared to monotherapy or vehicle treatment (Fig. 5F). Furthermore, IHC staining of the collected tumors revealed significantly decreased pS6 levels in the cohort treated with abemaciclib and trametinib even after treatment withdrawal (Fig. 5G-H).

The combination of CDK4/6 and MEK inhibitors was also tested in a patient suffering from a therapy-refractory anaplastic pleomorphic xanthoastrocytoma with a non-canonical *BRAF* mutation that had progressed after multiple surgeries, chemotherapy, and focal radiotherapy. This approach resulted in tumor regression followed by stable disease for two years on therapy and an additional 30 months of stable disease after treatment discontinuation. A full clinical summary of the patient is available in the supplementary section, along with a graphical representation and radiological images in Supplemental Fig. 4A-D.

In conclusion, combined treatment with CDK4/6 and MEK inhibitors showed superior effects in human-to-organoid transplants, patient-derived xenografts, and was active in one clinical case, leading to prolonged overall survival, reduced tumor growth, and delayed regrowth after treatment discontinuation. This effect is possibly mediated by reduced levels of phosphorylated S6 and inhibition of the PI3K/mTOR pathway, accompanied by a decreased mesenchymal phenotype.

## Discussion

*BRAFV600E* mutations characterize 5–15% of pHGG [2, 7], and concurrent *CDKN2A/B* alterations have been shown to further define tumors with dismal prognosis [35]. Although combined BRAF/MEK inhibition is approved for *BRAF*-altered pHGG, responses are often limited as tumors rapidly acquire resistance to targeted therapy through tumor heterogeneity, and cellular state transitions and tumors frequently show rebound growth after therapy discontinuation [2, 5, 7, 27]. To overcome the aforementioned limitations of combined BRAF/MEK inhibition, improved combinatorial regimens need to be developed. Given that additional genetic alterations can influence the response to BRAF and MEK inhibitor therapies, we hypothesized that *BRAF*-mutant pHGGs harboring a *CDKN2A/B* loss may be sensitive to combined MEK and CDK4/6 inhibition. Even though activating *BRAF*-alterations are present across a wide range of cancer types and confer sensitivity to MAPK pathway inhibition [36] the majority of available data is derived from melanoma, a malignancy with a well-characterized predilection for cerebral dissemination [37]. In contrast, clinical data in HGG patients remain limited. It is important to acknowledge that HGG and melanoma are biologically distinct diseases with different tumor microenvironments, cellular origins, and clinical behaviors, limiting direct comparisons. This distinction is further emphasized by the substantially higher mutational burden observed in melanoma compared to pHGG [38–40]. Nevertheless, both malignancies often share overlapping oncogenic co-mutations, and mechanistic insights from melanoma studies may provide a conceptual framework for guiding the translation and adaptation of these findings to HGG.

Our presented study highlights improved anti-tumor effects of simultaneous trametinib and abemaciclib treatment in preclinical *BRAF*-mutant pHGG models, particularly after therapeutic BRAF and MEK inhibition. These findings align with in vivo observations in *BRAF*-mutant melanoma, where uncontrolled proliferation and cell-cycle progression driven by *CDKN2A/B* deletion or *CCND1* overexpression/amplification have been implicated in resistance to BRAF inhibition [41, 42]. Furthermore, in a clinical study performed in metastatic melanoma, the patient subgroup characterized by alterations in these cell cycle-regulating genes showed shorter progression-free survival (PFS) with dabrafenib treatment [42] supporting our rationale for targeting MEK and CDK4/6 pathways in *CDKN2A/B*-altered *BRAF*-mutant HGGs.

Importantly, both groups of targeted compounds, CDK4/6 and MEK inhibitors, are known to induce cellular senescence [43–45], a phenomenon we also observed in our trametinib- or abemaciclib-treated *BRAF*-mutant HGG models. However, persistence of senescent cells during tumor treatment may have detrimental effects, including tumor cell invasion [46] or promotion of stemness features [47]. Thus, elimination of senescent cells by induction of cell death might overcome senescence-induced adverse effects. Indeed, we could demonstrate that the combination of trametinib and abemaciclib distinctly triggered cell death in a panel of HGG cells. This is in line with melanoma studies, showing that the combination of palbociclib and a MEK inhibitor exerts synergistic effects [48–50].

In our study, combined abemaciclib and trametinib treatment improved tumor responses in both spheroid and HOT models and demonstrated sustained anti-tumor activity after treatment discontinuation in a PDX, derived from a BRAF-inhibitor resistant tumor, compared to single-agent treatment. However, we observed rebound growth after therapy withdrawal paralleling studies in *BRAF*-mutant melanoma treated with palbociclib and a RAF (PLX4720) inhibitor [50] as well as in *NRAS*-mutant melanoma receiving palbociclib and a MEK inhibitor [51]. In contrast, in vitro and in vivo data in treatment-naive melanoma revealed that tumors did not acquire resistance to combined CDK4/6 and MEK inhibition and showed robust and rapid response after they were rechallenged upon tumor progression [50].

With respect to escape mechanisms following combined BRAF/MEK, upregulation of distinct RTKs has been described, including EGFR and PDGFR, as well as hyperactivation of MAPK, PI3K or cell cycle signaling cascades [2, 52]. Indeed, in our patient-derived VBT125 cell model established from a progressive lesion, treatment with dabrafenib and trametinib resulted in higher PDGFRA protein levels as compared to the matching treatment-naive primary model VBT92. However, avapritinib, a selective PDGFRA/KIT inhibitor with activity in PDGFRA-activated pHGG, did not exhibit single-agent activity in this model [53]. In addition, our data confirmed hyperactivation of PI3K/mTOR signaling, indicated by sustained S6 phosphorylation despite treatment with trametinib in the BRAF and MEK inhibitor-pretreated VBT125 model. These findings highlight the importance of the PI3K/mTOR pathway in resistance development to MEK and BRAF inhibition [54]. Notably, the combination of abemaciclib and trametinib resulted in profound suppression of S6 phosphorylation in both VBT92 and corresponding VBT125 cell models, indicating a potential therapeutic strategy to circumvent resistance mechanisms. This is corroborated by findings in *KRAS*-mutant colorectal carcinoma, where combinations of MEK and CDK4/6 inhibitors demonstrated synergistic antitumor activity and decreased phosphorylation of S6 [55].

In addition to acquired genetic alterations, plasticity in tumor cell states has been shown to widely influence the biological and clinical behavior of HGG [5], including *BRAFV600E*-mutant pHGG [27]. In this respect, the presented study demonstrated activation of hypoxia-responsive genes, a well-established driver of mesenchymal transition [34], suggesting that hypoxia-associated signaling may contribute to the enhanced migratory and invasive capacity observed in BRAFmut HGGs upon treatment with MEK and BRAF inhibitors. In accordance, treatment with dabrafenib and trametinib was associated with a marked increase in migratory potential, evidenced by enrichment of a CD44-positive cellular subpopulation. These tumor cells displayed a more elongated, spindle-shaped morphology, characteristic of the mesenchymal-like phenotype with aggressive and invasive growth capacity. To the best of our knowledge, a direct association between BRAF/MEK inhibition and the induction of a mesenchymal phenotype in pHGG has not yet been described. However, CD44-positive mesenchymal-like cells can be induced post radio- and chemotherapy in HGG [7, 56–58]. Based on our findings, we hypothesize that the combination of abemaciclib and trametinib exerts a sustained anti-proliferative effect by preventing the phenotypic shift of *BRAF*-mutant cells upon MAPK inhibition towards a mesenchymal state.

The translational relevance of our preclinical findings was substantiated by the clinical response of a patient with refractory anaplastic pleomorphic xanthoastrocytoma with a pathogenic *BRAF* mutation and CDKN2A/B loss. After multiple lines of standard treatment, therapy with ribociclib and trametinib resulted in initial response and tumor regression. Following two years of combined treatment, the patient maintained stable disease throughout the entire treatment period and for nearly 30 months thereafter without any therapeutic intervention. It is worth noting that for clinical reasons and drug availability, the combination of trametinib with ribociclib was selected, even though our preclinical data identified abemaciclib as most potent partner. While this underlines the necessity of further preclinical and clinical investigation, our clinical observation in pHGG is in line with experiences in *BRAF*-mutant melanoma patients where combinatorial treatment with a CDK4/6 and a BRAF inhibitor resulted in partial response in two and stable disease in six patients [59]. Similarly, *NRAS*-mutant melanoma patients with concurrent alterations in cell-cycle genes treated with ribociclib and binimetinib, showed a higher response rate when compared to wild-type counterparts [60]. In our clinical case, treatment was very well tolerated, with only mild adverse effects described for tyrosine kinase inhibitor therapy, such as weight gain, skin rash, and fatigue. This is in line with *NRAS*-mutant melanoma patients receiving a combination of ribociclib and binimetinib, where diarrhea, fatigue, and increased blood CPK levels were the most frequent adverse events [60]. In contrast, the combinatorial administration of trametinib and everolimus, targeting MEK and mTOR signaling, was clinically not well tolerated due to dose-limiting toxicities [61] or did not meet therapeutic goals due to rapid clearance [61–63].

## Conclusion

This study identifies combined CDK4/6 and MEK inhibition as a highly effective therapeutic strategy for *BRAF*-mutant, *CDKN2A/B*-deficient pediatric high-grade gliomas that overcomes key resistance mechanisms limiting the current targeted therapy with dual BRAF and MEK inhibition. The combination of trametinib and abemaciclib simultaneously suppresses PI3K/mTOR signaling and counteracts the mesenchymal-like cell state induced by MAPK inhibition. In addition, this combinatorial treatment induces cellular senescence and apoptosis, resulting in durable tumor control in human-to-organoid transplants, PDX models, and in a first translational clinical approach. In summary, our findings highlight the translational relevance of MEK–CDK4/6 co-targeting as a promising avenue for improving outcomes in therapy-refractory pHGG. Our study delivers a strong mechanistic rationale for combined CDK4/6 and MEK inhibition in *BRAF*-mutant, therapy-refractory patients, warranting further investigation in clinical studies.

## Supplementary Information

Additional file 1: Summary of the clinical case and Supplemental Figures 1-4.

Additional file 2: Supplemental tables; Table S1-list of primary antibodies for Western Blot; Table S2-list of primary antibodies for immunohistochemistry.

Additional file 3: list of differentially expressed genes.

Additional file 4: list of GO terms.

## Data Availability

RNA sequencing data from the R2 ITCC-P4 PDX Data Scope portal are available to the global scientific community upon access request. RNA sequencing data generated in-house from VBT92 and VBT125 will be uploaded to EGA upon publication.

## Abbreviations

pHGG: Pediatric high-grade glioma
CNS: Central nervous system
BRAF: v-raf murine sarcoma viral oncogene homolog B1
MEK: Mitogen-activated protein kinase kinase
MAPK: mitogen-activated protein kinase
BRAFi: BRAF inhibitor
MEKi: MEK inhibitor
HGG: High-grade glioma
aPXA: Anaplastic pleomorphic xanthoastrocytoma
GBM: Glioblastoma
WT: Wildtype
FCS: Fetal calf serum
RPMI: Roswell Park Memorial Institute medium
ATP: Adenosine triphosphate
IC_50_: Half maximal inhibitory concentration
DMSO: Dimethyl sulfoxide
PI: Propidium iodide
BBB: Blood–brain barrier
CDK: Cyclin-dependent kinase
CDK4/6i: CDK4/6 inhibitor
HPMC: Hydroxypropylmethylcellulose
HPC: Hydroxypropylcellulose
HEC: Hydroxyethyl cellulose
PBS: Phosphate-buffered saline
BSA: Bovine serum albumin
FFPE: Formalin-fixed paraffin-embedded
IHC: Immunohistochemistry
hESC: Human embryonic stem cell
GFP: Green fluorescent protein
ROI: Region of interest
PDX: Patient-derived xenograft
HOT: Human-to-organoid transplant
GSVA: Gene Set Variation Analysis
UMI: Unique molecular identifier
GEO: Gene Expression Omnibus
EMA: European Medicines Agency
FDA: Food and Drug Administration
OPC-like: Oligodendrocyte progenitor–like
NPC-like: Neural progenitor cell–like
AC-like: Astrocyte-like
MES-like: Mesenchymal-like
IRB: Institutional Review Board
EGA: European Genome–phenome Archive
HR: Hormone receptor
HER2: Human epidermal growth factor receptor 2
PEG: Polyethylene glycol
H&E: Hematoxylin and eosin
ITCC-P4: Innovative Therapies for Children with Cancer – Pediatric Preclinical Proof-of-Concept Platform
STAR: Spliced Transcripts Alignment to a Reference
PROGENy: Pathway RespOnsive GENes
FELASA: Federation of Laboratory Animal Science Associations
