# Supplementary material for: Co-targeting CDK4/6 and MEK reverses mesenchymal transition in therapy-refractory BRAF-altered pediatric high-grade glioma
Source: J Exp Clin Cancer Res. 2026 May 16;45:154. doi: 10.1186/s13046-026-03725-3 (PMC13348095; doi:10.1186/s13046-026-03725-3)
Supplement: Supplementary file 1 — Additional file 1: Summary of the clinical case and Supplemental Figures 1-4. [file 13046_2026_3725_MOESM1_ESM.docx]

**Additional file 1**

**Co-Targeting CDK4/6 and MEK Reverses Mesenchymal Transition in therapy-refractory BRAF-altered Pediatric High-Grade Glioma**

Mayr et al.

Clinical case and Supplemental Figures 1-4

Clinical Case Description

In a first translational approach, a patient with a treatment refractory anaplastic pleomorphic xanthoastrocytoma was successfully treated with ribociclib and trametinib. A previously healthy male (UMPED27) initially presented to the emergency room at age 5 with symptoms of nausea and vomiting. Magnetic resonance imaging (MRI) showed a large mass with a cystic component and no clear evidence of tumor infiltration. The patient underwent multiple partial resections, but minimal residual tumor remained along the anterior border. Histological diagnosis revealed an anaplastic pleomorphic xanthoastrocytoma, WHO grade 3 and next-generation sequencing showed non-classical BRAF mutation (p.L485_P490delinsF) and a homozygous CDKN2A/B loss. A full history of the patient's clinical course is depicted in Supplemental Figure 4A. Adjuvant chemotherapy with carboplatin and vincristine was administered but after the first cycle of maintenance therapy the tumor progressed locally and the patient underwent re-resection thirteen months after initial diagnosis (Supplemental Figure 4 B-C). The histopathological report remained unchanged and the postoperative MRI depicted a mild rim of enhancement at the anterior resection cavity consistent with residual tumor. Fifteen months from initial diagnosis he underwent focal photon radiotherapy with 59 Gy and Gamma Knife to a small distinct nodule in the septum pellucidum. MRI scans after radiotherapy revealed local tumor progression and due to the molecular profile, treatment with ribociclib (initiated with 400 mg daily for 21 days and increased to 500 mg afterwards due to good tolerability and to approximate 350mg/m2) and trametinib (initiated with 0.5mg daily and after 21 days increased to 1 mg corresponding to 0.025mg/kg/day) was initiated approximately 25 months from diagnosis. The tumor showed initial regression followed by stable disease and the patient tolerated the treatment well with mild side effects including weight gain, rash and fatigue. Treatment was administered for 24 months and MRI scans three months after treatment discontinuation showed a small increase of two local lesions followed by stable disease for almost 30 months.

Approximately 82months from diagnosis two tumor nodules were increasing in size and the patient underwent tumor resection of both lesions. Next-generation sequencing revealed a similar result with the same non-classical *BRAF* mutation and the homozygous *CDKN2A/B* loss, several new mutations with unknown significance and a *POT1* germline mutation (Supplemental Figure 4D) that was not covered in the gene panel of the initial analysis. This *POT1* mutation is associated with a tumor predisposition syndrome associated with increased risk of cutaneous melanoma, chronic lymphocytic leukemia, angiosarcoma and gliomas with unknown penetrance16 (Supplemental Figure 4D). Due to the extent of resection and stability of clinical symptoms, the family agreed to move to surveillance without further treatment, and the patient has remained stable at 96 months from diagnosis.

Supplemental Figures 1-4


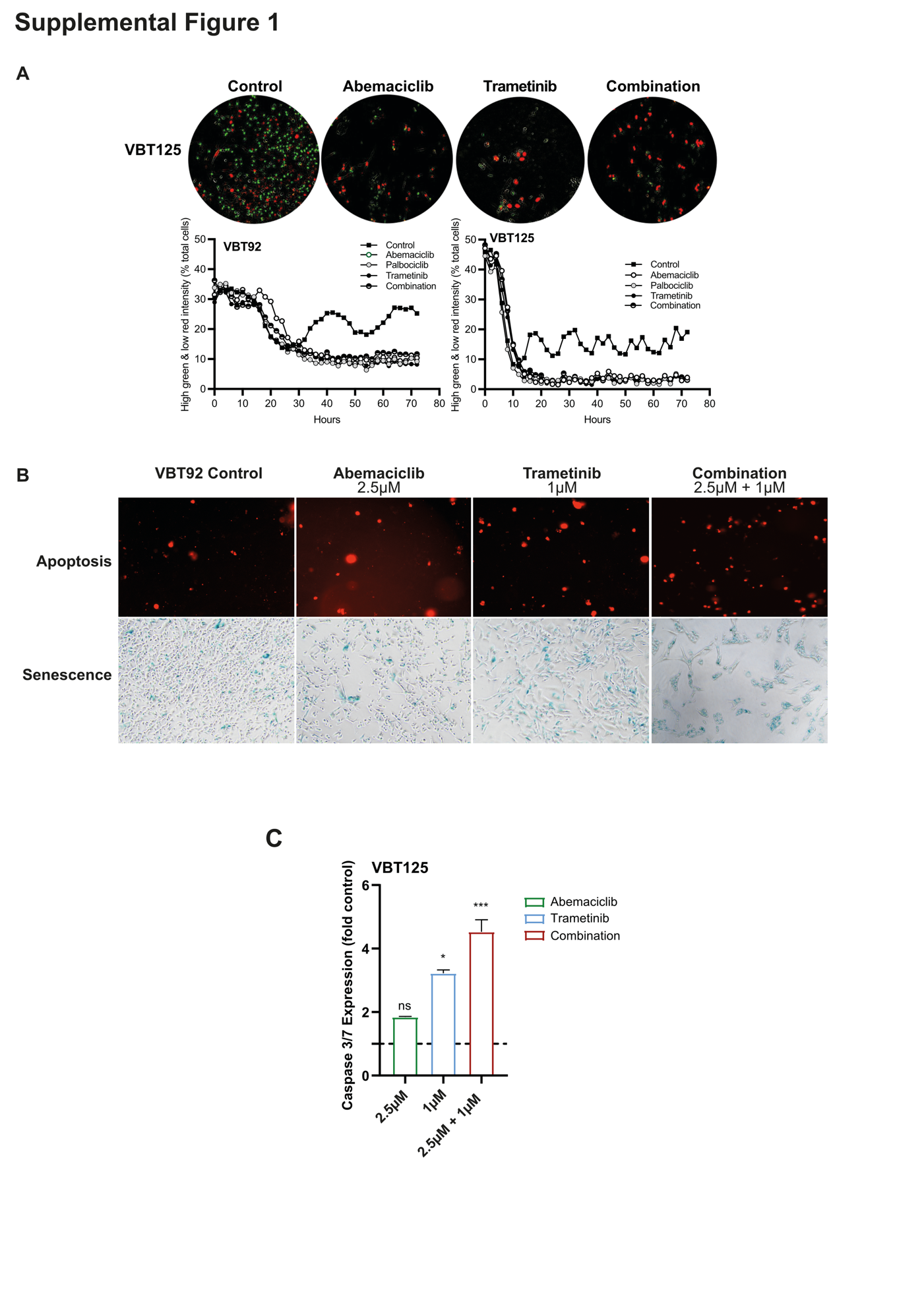


**Supplemental Figure S1. Induction of cell cycle arrest, apoptosis and senescence in BRAF-mut pHGG models** A. For cell cycle distribution analysis, respective images of VBT125 cells are shown. All cells were transfected with the Incucyte® Cell Cycle Green/Red Lentivirus Reagent and a thymidine block was applied twice to synchronize all cells. After the second release, cells were treated with 500 nM of the respective drug for monotherapy or 250 nM of the depicted drugs for combinatorial treatment. Cells were imaged in the Incucyte® Live-Cell imaging system for four days and cell cycle distribution was analyzed with the “cell-by-cell analysis” tool. B. Representative images from VBT92 24 hours after treatment are depicted for apoptosis in the upper and senescence in the lower panel. The corresponding data analysis is shown in Figure 2B. C. To assess the induction of apoptosis following mono-or combined treatment with CDK4/6 and MEK inhibitors, a caspase 3/7 assay was conducted using the Incucyte® Caspase-3/7 Green Dye and the Incucyte® live-cell imaging system. The assay was performed according to the manufacturer's instructions. Cells were treated with 2.5 μM abemaciclib, 1 μM trametinib or a combination of both inhibitors and imaged 24 hours after treatment. Unpaired t-test was used to determine statistical significance and the data is presented as mean +/- SD. Statistical significance is denoted as follows: p ≤ 0.05 = *, p ≤ 0.001 = ***.


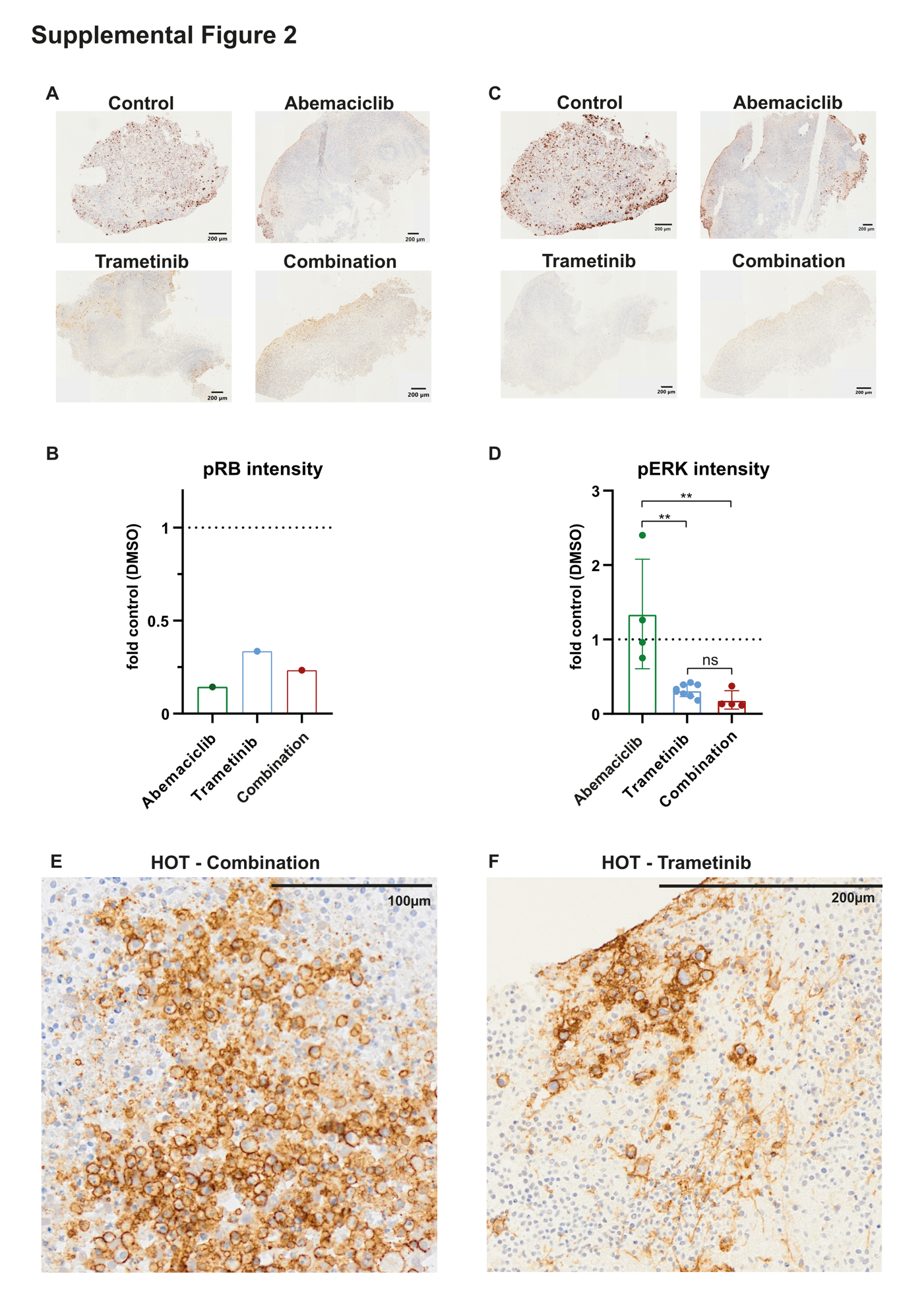


**Supplemental Figure S2. Target validation with immunohistochemical staining and quantitative analysis of pRB and pERK in pHGG HOTs.** A. Immunohistochemistry stainings of pRB were performed on the fixed HOT sections and slides were scanned with a slide scanner for digitization. Scale bars indicate 200 µM. B. Image analysis of the pictures in A was performed using ImageJ to calculate the mean staining intensity of pRB for one organoid each. C. Representative immunohistochemistry stainings of pERK are depicted for HOT sections. Slides were scanned with a slide scanner for digitization. Scale bars indicate 200 µM. D. Image analyses of regions of interest from two different organoid batches were performed using ImageJ to calculate the mean staining intensity of pERK in the tumor area of each organoid. E and F. Enlarged view of representative regions showing CD44-positive cells in HOT model following combination treatment (E) and treatment with trametinib (F). Scale bars indicate 100 µM and 200µM as depicted.


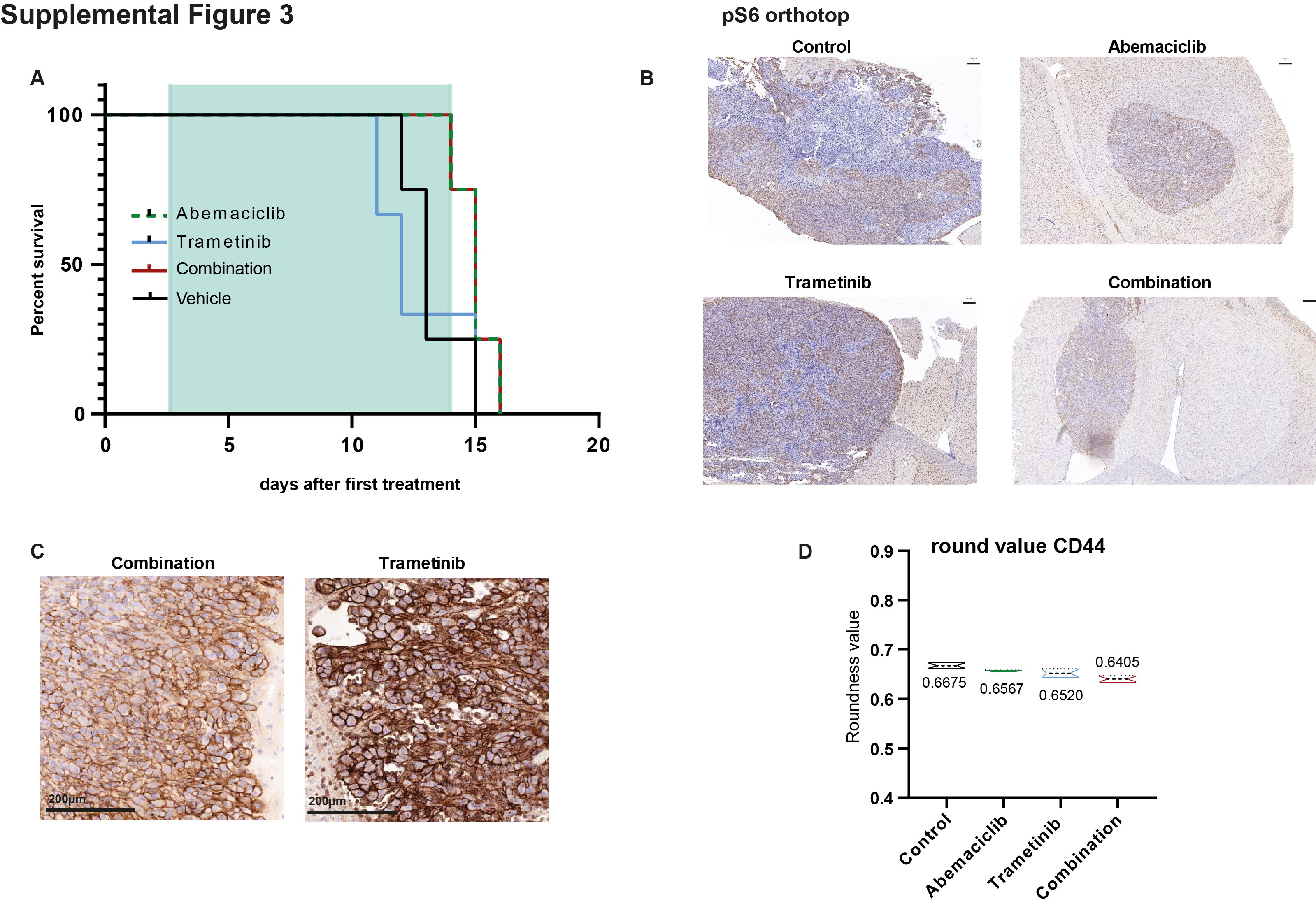


**Supplemental Figure S3. Effects of abemaciclib and trametinib on survival and PI3K downstream signaling in an** **orthotopic patient-derived xenograft model.** A. VBT125 cells were orthotopically implanted in NGS mice and treatment with a vehicle, abemaciclib, trametinib or their combination (n=4 per group) was initiated three days after injection for 14 days. Kaplan-Meier survival analysis was performed and the data is presented as mean +/- SD. B. Immunohistochemistry stainings of pS6 were performed on the fixed and stained orthotopic tumor sections and slides were scanned with a slide scanner for digitization. Scale bars indicate 200 µM. C. Enlarged view of a representative region showing CD44-positive cells in PDX models following combination (left) or trametinib (right) Treatment. D. Cellular shapes, expressed as roundness values (1=perfect circle; <1 more elongated shape), were quantified based on CD44-positive cells from orthotopic PDX tumors (2-3 tumors in each treatment group). Scale bars indicate 200 µM


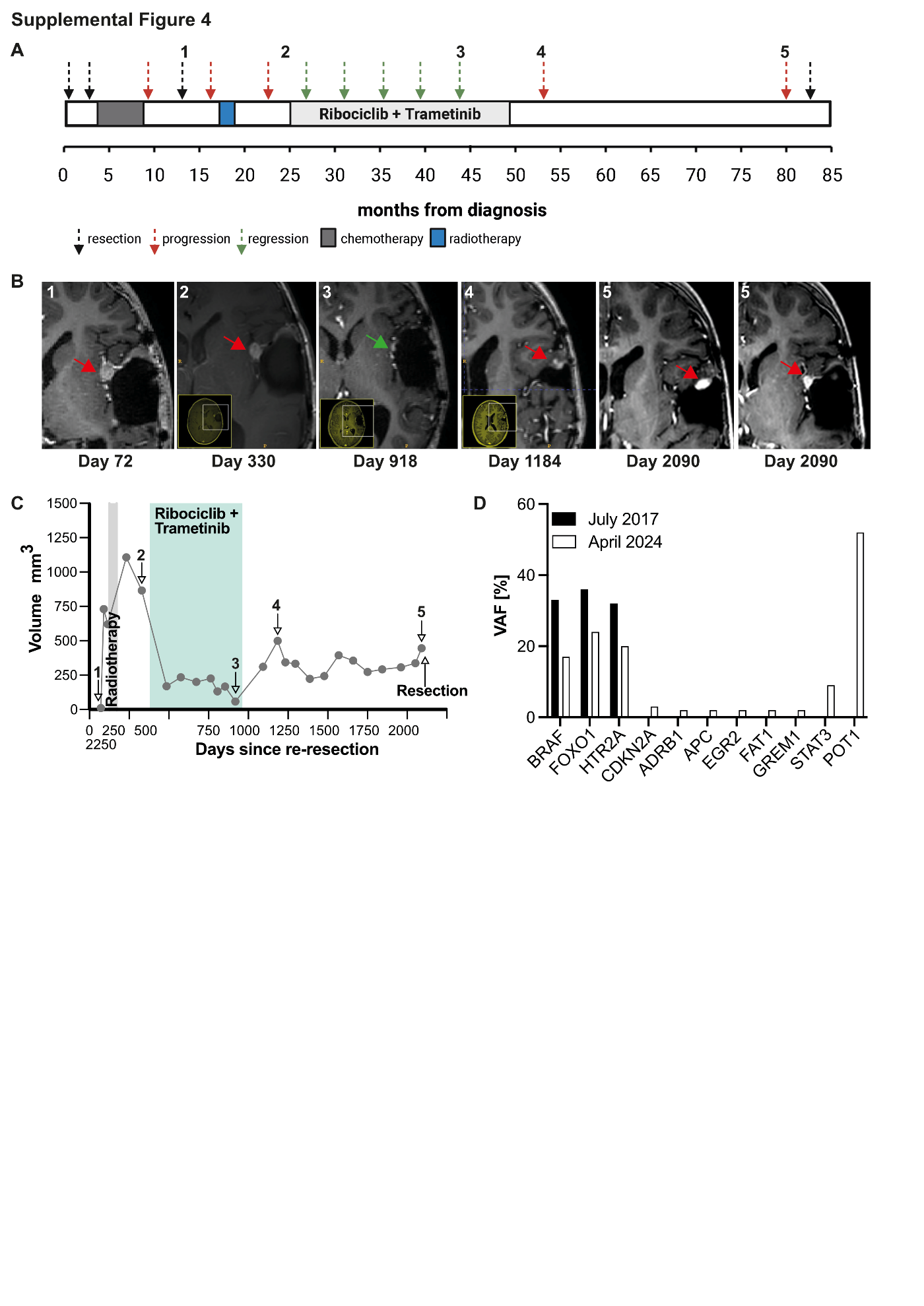


**Supplemental Figure S4. Clinical course of CDK4/6 and MEK inhibition in a refractory anaplastic pleomorphic xanthoastrocytoma patient.** A. Graphical timeline depicting the course of disease in our patient including the different administered treatment approaches. B. MRI images T1-weighted with contrast enhancing agent are depicted for the different timepoints starting after tumor re-resection. Red arrows mark progressive disease and green arrows response to treatment. The numbers depicted in the left upper corner of the images represent the time points marked in A and C with the respective numbers. C. Tumor volume was assessed of all available MRI images after re-resection and is depicted over the course of disease. The numbers displayed in the graph link the curve to the respective images in B (number in left upper corner). D. Differences in percentage of variant allele frequency of the tumor specimens analyzed with the OncoSeq assay from the primary tumor in 2017 and the progressing lesion after treatment with ribociclib and trametinib in 2024 are shown.
