# Supplementary material for: Co-targeting CDK4/6 and MEK reverses mesenchymal transition in therapy-refractory BRAF-altered pediatric high-grade glioma
Source: J Exp Clin Cancer Res. 2026 May 16;45:154. doi: 10.1186/s13046-026-03725-3 (PMC13348095; doi:10.1186/s13046-026-03725-3)
Supplement: Supplementary file 2 — Additional file 2: Supplemental tables; Table S1-list of primary antibodies for Western Blot; Table S2-list of primary antibodies for immunohistochemistry. [file 13046_2026_3725_MOESM2_ESM.docx]

**Additional file 2**

**Co-Targeting CDK4/6 and MEK Reverses Mesenchymal Transition in therapy-refractory BRAF-altered Pediatric High-Grade Glioma**

Mayr et al.

**Supplemental Table1-2**

**Supplemental Table S1. Primary Antibodies for Western Blot**

| pRB (Ser807/811) | Cell Signaling Technologies Cat#9308 | rabbit |
| --- | --- | --- |
| RB | Santa Cruz Cat#sc-73598 | mouse |
| pERK (Tyr202/204) | Cell Signaling Technologies Cat#9101 | rabbit |
| ERK p44/42 | Cell Signaling Technologies Cat#9102 | rabbit |
| pS6 (Ser240/244) | Cell Signaling Technologies Cat#2215 | rabbit |
| S6 | Cell Signaling Technologies Cat#2217 | rabbit |
| pAKT | Cell Signaling Technologies Cat#9271 | rabbit |
| AKT | Cell Signaling Technologies Cat#9272 | rabbit |
| EGFR | Cell Signaling Technologies Cat#2232 | rabbit |
| PDGFRA | Cell Signaling Technologies Cat #5241 | rabbit |
| CDK4 | Cell Signaling Technologies Cat#12790 | rabbit |
| CDK6 | Cell Signaling Technologies Cat #3136 | mouse |
| CD44 | Cell Signaling Technologies Cat #3570 | mouse |
| Beta Actin | Sigma Aldrich Cat#A2228 | rabbit |

**Supplemental Table S2. Primary Antibodies for Immunohistochemistry**

|  |  |  | pH | dilution |
| --- | --- | --- | --- | --- |
| pRB (Ser807/811) | Cell Signaling Technologies Cat#9308 | rabbit | 6 | 1:250 |
| pERK (Tyr202/204) | Cell Signaling Technologies Cat#4376 | rabbit | 9 | 1:500 |
| pS6 (Ser240/244) | Cell Signaling Technologies Cat#5364 | rabbit | 6 | 1:500 |
| CD44 | Cell Signaling Technologies Cat#3570 | mouse | 6 | 1:500 |
| GFP | Cell Signaling Technologies Cat#2555 | rabbit | 6 | 1:1000 |
